# "The Hospital" Nursing Mirror

**Published:** 1901-04-20

**Authors:** 


### "The Hospital" Nursing Mirror.

Being the Nursing Section of "The Hospital."

[Contributions for this Section of "The Hospital" should be addressed to the Editor, "The Hospital" Nursing Mirror, 28 & 29 Southampton Street Strand, London, W.C.]

### Notes on News from the Nursing World.

### QUEEN VICTORIA'S NURSES.

In the course of their appeal to the people of this country to adequately endow "Queen Victoria's Nurses," the Duke of Portland and his colleagues on the council, who will in future be appointed by Queen Alexandra, observe that if only sufficient funds were forthcoming no town or district in the United Kingdom would be without a nurse. This is the goal to be steadily kept in view, but it is obvious that in order to reach it there must be a large endowment fund to supplement the subscription list. As it is observed in the appeal, "The number of nurses would have to be multiplied at least three times before the council or their fellow-countrymen can feel that they have in any sense completed the Queen's work." This increase would mean an additional expenditure of at least £20,000 a year, which is a considerable sum; but those who regard the completion of the work as a sacred trust, and who are concerned to do honour to the memory of Queen Victoria in a practical manner, will not be slow to afford substantial assistance. We hope that the response to the appeal which has since been endorsed by the lady members of the Council, with Miss Florence Nightingale at their head, will be liberal enough to ensure a place for Queen Victoria's Nurses" among our national assets. A meeting will be held at Londonderry House, Park Lane on Monday, April 29th, at 4.30 p.m., in order to take steps for organised action throughout the country, and a fully representative attendance may be anticipated.

### LORD ROBERTS AND THE NURSING SISTERS.

In the course of a long South African despatch issued on Wednesday morning Lord Roberts pays a warm tribute to the services of nurses at the front. "I find it difficult," he says, "within the limits of a short paragraph to give expression to the deep feeling of gratitude with which the Nursing Sisterhood has inspired all ranks serving in South Africa. The devotion, skill, courage, and endurance displayed equally by the Army Nursing Service and by kindred organisations from the Colonies have excited my admiration, and fully justified the opinion I have held for years as to the necessity and economy to the service of an ample nursing service for our Army. Some of the nurses who have been the most helpful have been lent to the Army Nursing Reserve by the great hospitals in the United Kingdom. I propose, in a later despatch to bring to your notice the names of some of the most deserving." The names of the following nurses and Roman Catholic Sisters are, however, mentioned in the same despatch:—*Nursing Staff, St. Michael's Home, Bloemfontein.*—Mother Superior Frances Vernon. Sisters Annie, Caroline, Ella, Flora Elizabeth, Frances Mary, Frances Louise, and Isabel. Miss Edith Cotton, Miss Sophia Selene Jones, Miss Ricarda Kennedy, and Miss Elsa Orbanowska. *Roman Catholic Convent, Bloemfontein.*—Mother Superior Francis de Sales. Sisters Adrian, Alphonsus, Evangelista, Ignatius, Lucian, Magdalen, Mary Louisa, Melanie, Philomena, Raphael, St. Anne, St. Leopold, St. Louis, Stanislaus, and Teresa.

### THE PAY OF PLAGUE NURSES AT CAPETOWN.

We understand that the nurses on plague duty at Capetown are paid at the rate of £4 4s. per week, with quarters and rations. There is a great demand for them at this rate, and there is no doubt that some of the members of the Army Nursing Service Reserve who have lately been dispatched to South Africa will have the opportunity of gaining experience as plague nurses. The rate of payment seems liberal, but the duties are heavy, and, as the death of the matron of the hospital shows, there is an element of peril, which, however, is not at all likely to act as a deterrent.

### A CRY FROM NO. 13 STATIONARY HOSPITAL, PINE TOWN BRIDGE.

"This is nothing more nor less than a depot where soldiers who have been very ill further up country are sent down to wait for the next boat that has come to take them to England," writes a sister in charge. "Now the waiting time is long to many, and though, of course, we are well supplied with necessary things yet we need a few extras badly, just to keep the hearts of these lads soft, for nothing touches them so much as a little bit of unexpected kindness. Books, games, papers, and a few shirts, socks, and handkerchiefs would be most acceptable. Just at present we have from Delagoa Bay a large batch of men with malaria, which comes on very suddenly. As I walk round I find those I left quite well a little while before in bed shivering, with extra blankets on; all they say is, 'I've got a touch of it again, Sister.' I brought some games out with me, but they are all gone; for Tommy likes to lay hold of his small plum, and does not think of the next comer. I feel sure that some of the readers of 'The Hospital' Nursing Mirror will help us to cheer and brighten the lot of the men who came willingly to fight for their country, leaving their nearest and dearest for the sake of duty, and send them back to England with the recollections of hardships forgotten."

### THE CONGRESS OF NURSES AT BUFFALO.

As there seems to be an erroneous impression that the official head of the coming Congress of Nurses at Buffalo is an English lady, we may repeat the statement that the president and chairman of the Congress is Miss Isabel McIsaac, of the Illinois Training School, Chicago. The first vice-presidents are Miss Robb and Miss Keating, and the second vice-presidents Miss Damer and Miss Snively. We may add that it has been decided to ask Miss Florence Nightingale to accept an honorary title, and that representative nurses of the different countries shall be placed on the list of honorary officers.

### A PRIVATE NURSING INSTITUTE FOR HONG KONG.

It has been decided to form a private nursing institute in Hong Kong. At a meeting held last month in the City Hall of the colony, at which Lady Blake, wife of the Governor, and Bishop Hoare were among those present, Dr. Stedman explained the reasons for starting the organisations, and the nature of the scheme itself, which is practically that adopted by the Colonial Nursing Association. In other words, an influential and representative committee of both sexes is to manage the institution, to receive the fees earned by the nurses, and to pay them a fixed annual salary, providing also for their board and lodging. Some difficulty was experienced in finding a place of residence for the nurses, but eventually it was found practicable to allow them, for a year at any rate, to have rooms at the new Peak Hospital. They will not, of course, be called upon to do duty at the Peak Hospital, but they will have the advantage of being able to associate with the hospital nurses, while the building itself is close to the tramway and very central both for the town and for the Peak. It is proposed to start with two nurses, and to offer a sufficient salary to render the appointments attractive; and it is hoped that the Government will render some financial assistance. But, of course, the colonists are depending for support mainly upon subscriptions, and upwards of 3,000 dollars were promised at the meeting.

### NURSING IN THE "HERMIT KINGDOM."

The conditions under which nursing is carried on in Corea, as described in another column, are not ideal in the only existing hospital, while, as our correspondent says, "nursing in a Corean house is heart-breaking work." Nevertheless, it is distinctly encouraging to learn that the native patients appreciate good nursing. When this is the case there is always hope that the situation will improve. Corea is just now regarded with especial interest owing to the statement that Japan and Russia are likely to go to war for possession of the country. The statement, it may be hoped, is an exaggeration, but there is no doubt that the "Hermit Kingdom" has many natural advantages which have not escaped the attention of foreigners, and that nursing, like many other things, is only in its infancy at present.

### THE NEW HEAD NURSE OF THE GENERAL HOSPITAL, BARBADOS.

A new head nurse has been appointed to the General Hospital, Barbados, in the person of Miss Agnes Veecock, a daughter of the late Mr. J. Veecock, accountant of the Supreme Court of British Guiana. Miss Veecock was trained at the Royal Infirmary, Edinburgh, and after completing her three years' course entered the Simpson Memorial Hospital to go through a maternity course, in which she obtained a first prize. It may be hoped that the new head nurse will not find the difficulties of her position insuperable.

### A CASE FOR INQUIRY BY MR. WALTER LONG.

The communication which we print in another column, signed "Deeply Interested," is from a lady who herself occupies the position of superintendent nurse in a very important infirmary. We cordially endorse her hope that nurses who are qualified to fill the post of superintendent nurse at Croydon Workhouse Infirmary will refrain from applying for it. Miss Julian deserves the utmost practical sympathy and support, because she is fighting for a principle of the first importance. She and our correspondent "Deeply Interested" are doubtless only two among many matrons and superintendents of workhouse infirmaries who have found their efforts to weed out unsuitable candidates for training frustrated, and whose refusal to sign certificates of persons who have proved incapable nurses should, in the interests of the vocation, be strongly backed up. We agree that the attitude of the Local Government Board in the Croydon case is difficult to understand, and we ask Mr. Walter Long to personally inquire into a matter which affects trained nurses in all parts of the country. He has stamped out hydrophobia; it will be gratifying if he assists to stamp out incapable nurses in workhouse infirmaries.

### THE NURSING OF CASES OF PHTHISIS.

The lady superintendent of the Hospital for Consumption and Diseases of the Chest was asked by our commissioner, whose account of an interview with her appears in another column, whether she considered that there is any risk of contagion in the nursing of cases of phthisis. Her answer was, "Practically none," and she went on to say that, while every possible precaution is taken in the way of disinfection and fresh air, "the nurses who have been in the hospital for so many years are themselves a proof that there is no risk." They are certainly a proof of the care that is taken to guard against contagion. As in the nursing of cases of typhoid, so in the nursing of those of phthisis, almost everything depends upon the nurses themselves. At the Brompton Hospital every inducement appears to be offered to render the work of nursing patients suffering from consumption as attractive and as safe as possible.

### THE EAST LONDON NURSING SOCIETY.

The annual meeting on behalf of the East London Nursing Society will be held at the Mansion House on Tuesday afternoon, and, as usual, the matron and nurses will be present. On Friday morning, May 10th, there will be a special service at St. Paul's Cathedral, with a sermon by the Bishop of Barking. The report of the committee of the Society shows that during last year upwards of 5,000 cases were nursed, 782 of the cases being those who were thus helped to regain strength, and with it the power of maintaining themselves and their families. It is an interesting fact that five more of the staff—Nurses Beer, Busselle, Gibbs, Pople, and Toole—have been admitted into the ranks of the Queen's Nurses. Unfortunately, the income was not sufficient for any extension of operations, and but for the kindness of the Lord Mayor and Corporation and the London Parochial Charities the committee would not have been able to maintain their present staff. The population among whom the staff of the East London Society carry on their arduous work of mercy is 337,800, and there are twenty-eight districts, or thirty-four parishes, in which a trained nurse is employed. The expense of maintaining each is £60 to £70 a year, according to length of service and cost of furnished lodgings.

### THE STEPNEY SCANDAL.

We learn with much surprise that the Stepney Board of Guardians have not only taken no measures in respect to the two assistant nurses who were accused at a coroner's inquest of neglecting a woman in one of the infirm wards of the workhouse, but have also refrained from making any change in the nursing staff, thus entirely ignoring both the opinion expressed by the sister superintendent and the doctor, and the evidence given in the coroner's court. The result of the inquiry with closed doors would therefore appear to be *nil*. If, in such circumstances, anything untoward happens to any of the acute cases that are taken into the wards, when they cannot be received into the sick asylum "across the way," the Guardians must expect to be censured.

### THE NURSING VOCATION IN AUSTRALASIA.

In an article entitled "Hail the Nurse!" the *Australian Medical Gazette* deals severely alike with the fiction-mongers who, taking their cue from the popular belief, indulge in gross caricatures of nurses, and with the prejudiced people who are guilty of the sneer that women enter the hospitals for matrimonial purposes. Maintaining that "perhaps the only true instance in the world of *camaraderie* on a platonic basis is that which exists between the doctor and the nurse, the writer concludes with a tribute to nurses, which shows that at the Antipodes no less than in the old country the vocation of the latter is worthily upheld. "The trained nurse is undoubtedly one of the most valuable products of our civilisation. She is the servant and minister of all, ready, like the fireman, to go off anywhere at a moment's notice, to sink personal convenience and comfort, to undertake a five hundred mile journey into the bush, or face a case of diphtheria or plague. If the general public will not realise the silent and unsung heroism to be found in the lives of so many of these women, let us of the medical profession who have eyes to see not withhold from them their meed of praise."

### QUEEN'S NURSES AT TAUNTON.

The Taunton District Nursing Association has been affiliated to Queen Victoria's Jubilee Institute for Nurses, and it has thus followed the example of Bath, Bridgewater, and Chard. The advantages of the arrangement which has been rather tardily made, considering that Taunton is the county town of Somerset, have been repeatedly pointed out, and we are glad to see that the local press attach importance to the systematic inspection of nursing by a qualified representative from headquarters, which is ensured under it. This inspection, which we believe is done thoroughly, is valuable for many reasons. It helps to put both the committee and the nurses of an association on their mettle, gives a new interest to the work, and also places experience at the disposal of the organisation which could not otherwise be available.

### KEEPING TO THE RIGHT PRINCIPLE AT BOLTON.

We are glad to see in the report of the Bolton District Nursing Association for 1900 a notification that "the nurses are not, under any circumstances, allowed to receive gratuities from the patients or their friends, but patients are encouraged, when able, to give donations, however small, to the general fund." Last year £19 was thus contributed. This is the right principle from which there should be no departure on the part of those who are engaged in supplying district nurses for work among the poor. Any attempt to combine gratuitous nursing of the poor with paying patients of another class, is not in accordance with the objects for which the Queen Victoria Jubilee Institute was founded. The superintendent at Bolton and her staff of ten nursed in the twelve months 1,417 cases, of which 882 became convalescent.

### STAFFORDSHIRE INSTITUTION FOR NURSES.

The committee of the Staffordshire Nursing Institution are again able to report continuous employment, large earnings, and approved work. The staff for 1900 consisted of 141 nurses, of which 92 were private, 18 district nurses, and 31 probationers. The cases nursed were fewer in number than in 1899, but the duration of the cases was longer, and altogether the nurses gave 3,849 weeks of nursing. The earnings reached the highest figure yet attained—£5,871. Of the 832 cases reduced fees were accepted in 12, and the services of the nurses were given gratuitously in 13 instances—£105 17s. being drawn from the "Sick Poor Nursing Fund" to meet the deficit in fees. The subscriptions and donations to this fund for the year have been £72 18s. 8d., leaving a balance in the bank of £56 1s. 4d. after all expenses have been paid, whilst the sum to the credit of the institution itself stands at £1,100. This is after dividing amongst the nurses £776 7s. 1d. as bonus percentage on their earnings for 1899.

### ST. GEORGE'S INFIRMARY, FULHAM ROAD.

The usual yearly examination of probationers was held at this infirmary during the first week in April. Thirteen probationers passed the written and practical examination in a very satisfactory manner and obtained certificates. The examiner was Dr. Burnard Neal, of the Wandsworth and Clapham Infirmary.

### IS THIS A RECORD?

At the annual meeting of the Stockton-on-Tees Nursing Association it was announced that during the year which the report covered a death occurred of a patient whom the nurses had attended for nearly nine years, and had visited 972 times. It was also stated that out of the 624 other cases nursed during the year no fewer than 617 were sent in by the local medical practitioners, thus showing the confidence placed by the medical faculty in the lady superintendent and her staff of assistants.

### SHORT ITEMS.

The lady superintendent and nurses of the Royal Derby and Derbyshire Nursing and Sanitary Association have had the honour of receiving acknowledgements from the King and Queen for the addresses of sympathy presented to their Majesties in respect to the death of the late Queen Victoria.—A café-chantant in aid of the District Nursing Home and Maternity Charity, Plaistow, will be held on May 21st at 24 Park Lane.—Two persons are reported to have died at Lariboisière Hospital, Paris, owing to the result of the mistake by a nurse who administered chloride of zinc to seven patients. The other five are said to be out of danger.—The annual meeting of the Zenana Bible and Medical Mission will be held on Wednesday afternoon in St. James's Hall, Piccadilly.—A feature of the new Richmond Surgical Hospital at Dublin, which will be opened on Saturday by Lord Cadogan, is the handsome nurses' home, of which details have already been given in our columns.—Nursing Sister S. A. Fisher, who left for South Africa in the hospital ship *Avoca*, was trained at the Adelaide Hospital, Peter Street, Dublin, and was appointed staff nurse in the South-Western Fever Hospital, Fulham. Thence she proceeded to the Royal Victoria Hospital, Netley, and was one of the first of the Army Nursing Reserve who volunteered for active service. While in South Africa she was attached to No. 6 General Hospital, Naauwpoort, where she contracted enteric fever and was invalided home. Prior to her departure again for South Africa she had been doing duty at Portobello Military Hospital.—The sum of £8,000 is required for the erection of the nurses' home at the new Somerset Hospital, Capetown. Towards this amount the Government has contributed £4,000, and the large firms in Capetown £2,000.—A lecture will be given on Thursday next by Mr. Richard Greene at the home of the Royal British Nurses' Association, 10 Orchard Street, W., at 5.30 p.m., on "Ancient Medicine and Ancient Medical Men."

### Medical Lectures to Nurses.

By CARSTAIRS DOUGLAS, M.D., B.Sc., F.R.S. Edin., F.F.P.S. Glasgow, Professor of Medical Jurisprudence, Anderson's College Medical School, and Dispensary Physician, Samaritan Hospital, Glasgow.

EVERY case of poisoning is to be regarded as an emergency. No doubt a considerable number recover, but one can never tell, when a case presents itself, what the termination may be. The effect of a poison is determined by many different factors, such as the amount swallowed, the solubility of the poison, the age, state of health and idiosyncrasy of the patient, the presence or absence of food in the stomach, and so on. It is hardly necessary for a nurse to disturb her mind with a consideration of all these points when face to face with an emergency case; what she must keep in mind are—(1) the symptoms produced by the more commonly employed poisons, and (2) the antidotes suitable for each. In many a medical case where a nurse is engaged, she can, at her leisure, read up the subject and prepare herself for any sudden complication that may occur; in poisoning cases, on the other hand, she must have her knowledge ready for immediate use, for she never knows when it may be needed. The great majority of cases of poisoning occur quite suddenly as the result of gross carelessness, and many a useful life has been lost because no one was at hand with the requisite knowledge and skill.

In the brief space at our disposal we can only consider a few of the leading poisons, with the symptoms produced by them, and their appropriate treatment. In not a few cases it is impossible to say at first hand what substance has been taken, and a nurse must be prepared to sacrifice accuracy of diagnosis for promptitude in action. What we aim at in all cases are: (1) the neutralising of the poison swallowed; (2) its removal from the body; (3) the counteracting of the effects of that portion absorbed; and (4) the general care of the patient.

*Carbolic Acid.*—The solid acid occurs as pinkish deliquescent crystals, with a penetrating typical odour. Readily soluble in water to form clear solutions; cheaper preparations are often dark. Immediately on swallowing there is felt a burning pain: giddiness ensues with collapse, a pale face, and cold skin. The breathing is stertorous, the pulse rapid and feeble. The urine is scanty and dark. Death may occur in from one to four hours. The lips and gums are softened and white, and the breath smells of carbolic acid. In treatment the stomach may be washed out with the rubber syphon tube; emetics do not act well. The best antidote is half an ounce of Epsom salts in half a tumbler of water, repeated in half an hour. In addition white of egg beat up with milk, olive oil and similar demulcent substances to soothe the burnt surfaces. The patient must be kept thoroughly warm, and an enema of brandy and bovril may be given.

*Oxalic Acid.*—This common poison is met with solid as slender prismatic crystals readily soluble in water. Immediately on taking it a burning sharply acid taste is felt, followed by pain in the throat and stomach. Shortly after, vomiting occurs, of food, acid fluid and mucus; there are severe abdominal cramps, purging, perhaps, and always collapse. The skin is moist and cold and the pulse feeble. In some cases the patient is drowsy. Death may occur in from two to twenty-four hours. As an antidote chalk is one of the best. Powdered chalk or whiting stirred up in a little milk or water, chalk mixture, or lime-water may be freely given. Calcined magnesia in milk is also good. Do not give preparations of soda or potash, and cut down the amount of liquid swallowed. Follow up with a good dose of castor oil. Maintain the patient's strength.

*Arsenic*, or, more correctly, arsenious acid. Practically tasteless. No symptoms for about an hour; then violent burning pain in the stomach and severe vomiting often of mucus or blood. Purging and griping pains in abdomen and copious watery motions. Great general collapse, with sunken features and cold clammy skin. The best antidote is ferric oxide prepared fresh. To do so take, say, an ounce of steel drops (ferric chloride), mix with water, and add some ammonia. A reddish-brown precipitate forms which should be strained off, washed, and administered stirred up in water. Next best is calcined magnesia stirred up in milk. Olive oil and demulcents soothe the stomach: a hypodermic of morphia may be needed for pain.

*Perchloride of Mercury*, or corrosive sublimate, is not uncommonly a cause of poisoning. Its action is similar to that of arsenic, though not so violent. An efficient antidote is found in the whites of eggs, four or five of which may be mixed with milk and given. Any albuminous substance, such as barley-water, may be similarly used.

*Phosphorus.*—Cases may arise from children sucking the heads of "strike-anywhere" matches. Pain is felt in the stomach; vomiting is urgent, the vomited matter smelling of phosphorus and being luminous in the dark. The breath smells of garlic, and there is a great thirst. In treatment three or four grains of copper sulphate dissolved in water should be given and repeated in half an hour. In its absence, a teaspoonful of Sanitas fluid is useful. Oily substances must be avoided. Ice and demulcent drinks will soothe the stomach.

*Opium.*—This, as morphia, laudanum, or some other preparation, is a very common poison. In a large dose it leads to drowsiness, stupor, and eventually complete insensibility, from which the patient can hardly be roused. The skin is moist, the face very pale, the breathing slow and puffing, and the pulse slow and full. A noticeable feature is the extreme contraction of the pupils. In treating a case, the stomach tube should be passed and all poison removed if possible. One of the best antidotes is potassium permanganate which can be introduced into the stomach as small doses of Condy's fluid. The skin should be slapped with wet towels, a strong faradic current applied to the muscles, and the patient walked about if possible. A hypodermic injection of a twentieth of a grain of atropine may be given, and an enema of half a pint of hot coffee to stimulate the patient.

In conclusion it may be mentioned that the best emetics are (1) Zinc sulphate, 20 grs., in a tumbler of warm water; (2) a tablespoonful of mustard given in the same way; (3) copper sulphate in 3 gr. doses also in warm water; and (4) where the person cannot swallow, $\frac{1}{10}$ gr. of apomorphine hypodermically.

### To Nurses.

**WE invite contributions from any of our readers, and shall be glad to pay for "Notes on News from the Nursing World," or for articles describing nursing experiences, or dealing with any nursing question from an original point of view. The minimum payment for contributions is 5s., but we welcome interesting contributions of a column, or a page, in length. It may be added that notices of entertainments, presentations, and deaths are not paid for, but, of course, we are always glad to receive them. All rejected manuscripts are returned in due course, and all payments for manuscripts used are made as early as possible after the beginning of each quarter.**

### The Nurses of the Hospital for Consumption and Diseases of the Chest.

By Our Commissioner.

### A CHAT WITH THE LADY SUPERINTENDENT.

Before I asked the Lady Superintendent of the Hospital for Consumption and Diseases of the Chest, at Brompton, any questions, Mrs. Price courteously suggested that I might like to see the Nurses' Home, and we accordingly traversed the subways which connect the new with the old part of the building. A journey through these labyrinthian passages gives an excellent idea of the extent and value of the site of the institution, which, whether on the ancient or the modern side, is full of interest. The former, for example, includes the artistic hall and the handsome chapel, with its beautiful coloured glass; while the galleries of the lofty wards are a feature of the latter. We paused for a minute or two in one ward where the open-air system is in operation, and, though the afternoon was not cold, I confess that I did not desire to prolong my stay indefinitely.

### The Nurses' Home.

But I can well understand the nurses of the hospital being greatly attached to the charming home, which was opened last June and possesses everything that could possibly be desired, with one exception. In the library there is a bookcase which is only half filled, and great, I doubt not, would be the delight of the lady superintendent and her staff if some kind friend found occupants for the empty shelves—not musty tomes, but useful works of reference and readable novels. For the rest, no want appears to have been neglected, and an immense amount of pains must have been taken not only to provide for the comfort of the inmates, but also to render the appearance of the entire home attractive. There is evidence of taste everywhere, and also of originality, particularly in the shape and furnishing of the principal rooms, which, instead of being monotonously alike, are all delightfully different. The capacious apartment sacred to the staff nurses contains, as well as many lounge and rocking chairs, a grand piano; the smaller one, belonging to the probationers, a cottage piano.

"But the sisters," it may be interjected. Well, the home sister, who is in charge of the home, and the night sister have a very nice room of their own, but the rooms of the rest of the sisters are in the hospital. With a glance at the very large dining-room, the kitchen, which is entirely for the use of the servants employed in the home, and the snug waiting room in which the nurses can see their friends, we proceeded to the next floor.

### The Bedrooms.

Here the Lady Superintendent pointed out a box room and four bath-rooms.

"There are the same on each of the other two floors," she continued, "and each nurse has a bedroom to herself, containing a fire-place."

"And very pretty and ample furniture," I said as I looked round me, and observed a wardrobe with a mirror in the door, a dressing table and chest of drawers combined, a marble-topped washing-stand with a tiled back, a small hanging bookshelf and cupboard.

"How many bedrooms are there altogether?" I inquired.

"Ninety-six," was the rejoinder, "and every one of them is furnished in the same manner. As you see the rooms for the night nurses are entirely shut off, and in the Home, as well as in the hospital, the electric light and lifts contribute to convenience and the saving of trouble. The fourth floor is occupied by the servants."

The Home includes provision for the private nursing staff, under the charge of a sister, who have, however, a separate sitting-room and dining-room, and are quite distinct from the hospital staff, the kitchen alone being used for both.

### The Staff and the Training.

"And what is the full strength of the hospital staff?" I said when we got back to the Lady Superintendent's own office.

"There are seven ward sisters, two night sisters, a home sister, an out-patient sister, a sister in charge of the private nursing branch, and a housekeeper who is a trained nurse. The staff nurses and probationers now number sixty."

"Has the number been augmented lately?"

"Yes; since last June five night nurses have been added, and some probationers. There are twenty-eight nurses on the private staff, all, of course, fully trained."

"Will you tell me about the training of your probationers?"

"We train for three years, but for the second year the probationers are sent to a general hospital or infirmary. Some go to Guy's, others to St. Thomas's, and Marylebone. There is no doubt that this arrangement is more satisfactory than if the training were confined to our own hospital."

"What lectures are given here?"

"The chief resident medical officer and myself deliver two courses, and the home sister holds classes once a week. During the third year a course of lectures is given by one of the physicians of the staff."

### Uniform and Off Duty.

"How are the probationers paid?"

"They receive £10 the first year, £12 the second, and £24 the third. After that their salary as staff nurses rises to £30. Indoor and outdoor uniforms are provided."

"Is it compulsory to wear it out of doors?"

"I like the nurses to wear it out of doors when they are only away from the hospital for an hour or two. They are quite at liberty to discard it when they are away for the day."

"How often do they get a day off?"

"Once a month. This applies to all nurses. The nurses and probationers also have two hours one day, and three hours the next, right through the week. The staff nurses, have from 5 to 10 off one evening in the week; and they have from 4.30 to 10 on alternate Sundays. They have likewise three weeks' holiday in the year. All the nurses are expected to attend service in the chapel on Sunday, either in the morning or the evening."

"Do the sisters get much more time off duty?"

"They have two hours and a half off every day, one evening in the week, as well as one afternoon and part of the evening; and every other Sunday from 4 p.m. They also have a whole day off once a month, and a month's holiday. A sister is in charge of a gallery with 40 beds in small wards containing from one to eight."

"When do the night nurses go on duty?"

"At nine, and they go off at eight. They have a night off once a month. There are two night sisters—one for each building. The nurses are on night duty for four months in the year. No probationers are assigned night duty."

### The Private Staff.

"Do you train most of your private staff?"

"Nearly all. A few come from other hospitals. Some of my old staff at Liverpool have followed me to Brompton. If at the end of the three years here a nurse goes on to the private staff, she receives £28; this is increased by degrees to £40, indoor and outdoor uniform being provided. A month's holiday is allowed to each nurse."

"What rest is permitted between cases?"

"If a nurse has had a long case, I like her to have a rest of two or three days, and to go away if possible; but sometimes between short cases there is no opportunity for a break. We cannot make a hard-and-fast rule."

"Most of the applications for nurses are for chest cases, I suppose?"

"Yes; during the last twelve or eighteen months I have had several for cases of out-door treatment. The nurses are specially trained for that purpose. In my opinion open-air treatment is decidedly becoming more general."

"Do you often have cases of illness among the nurses?"

"No; but if they are ill they are well looked after here, whether they are hospital or private nurses. In fact, everything is done that can be done to make them comfortable."

THE QUESTION OF CONTAGION.

"Is there any risk of contagion to nurses?"

"Practically none. Every possible precaution is taken in the way of disinfection and fresh air. The nurses who have been in the hospital for so many years are themselves a proof that there is no risk. A sister who lately retired was here for twenty-eight years. One is still with us who has been twenty years, and three have been over fifteen. There has not been a case of phthisis among the staff since I came two years ago."

"You were previously matron at Mill Road Infirmary, Liverpool?"

"Yes, I was matron for three years, and before that I was sister at Guy's Hospital for some years. I was trained at Guy's.

"Have you any age limit in operation?"

"No. Probationers are admitted at twenty-two, though I prefer twenty-three or twenty-four. I regret that so much is being made of the age question in the nursing world, and I think it should be more a matter of capacity than of age."

THE PENSION RULES.

"We have, as you know," continued the Lady Superintendent, "for several years been affiliated to the Royal National Pension Fund for Nurses."

"Do you find that many of the staff avail themselves of the arrangement?"

"A fair number of the nurses belong to it, but some who are very young will not join. They will probably change their minds later on. So far, none of our nurses has received a pension. I do not know that there are any special features in our pension rules. One rule is that the hospital annuity shall not exceed the amount of the annuity assured by the nominee's own policy, and in any case the limit is £19 10s. for a sister and £13 for a nurse, and another that no hospital nominee has any claim under her hospital policy unless she has been for five continuous years in the service of this hospital, or of some other hospital approved by our committee.

And then, after a stroll round the grounds, where many of the patients were taking exercise, I left with a conviction that, all things considered, the lines of the nurses of the Hospital for Consumption in Fulham Road have fallen in pleasant places.

### Typical Patients.

### THE UNLOVED BABY.

HIS name was Daniel Daly, but we called him Dolliwag. He came to the hospital many times as an "out-patient," carried in a bundle of dirty rags by his deplorable mother, who despised and neglected him. He had a wizen and wrinkled face with big black beady eyes, staring wonderingly, piteously, at every object they beheld, and never closing, for he was never known to sleep like other babies. His tiny body was so thin that each rib was distinctly visible, and his tinier limbs were cramped and rickety. It is useless to describe the misery he personated, but it seemed impossible that such a being should be called a child of a civilised race. Yet it is true that we shut our eyes to thousands of just such miserable babies in all our towns at the present time.

His mother evidently brought him to the hospital merely for the sake of begging the doctors to take him in, that she might be rid of an undesired burden and not for the sake of the kindly advice and medicine she received, because his body grew smaller and his eyes larger week by week. But, because the cots were sorely needed for other children who were not only suffering from neglect, Dolliwag was not admitted until he developed pneumonia.

It was a bitterly cold day when his mother, looking even more degraded than usual, brought him into the doctor's presence with a great flourish, and proceeded to unroll his rags triumphantly.

"'Es got a 'orful weezing on 'is chest," she said. "Yer can't send him out in this 'ere wind—it 'ud kill 'im."

After putting his stethoscope to the wee body the doctor with a sigh signed an admission paper, and handed it to me, telling me to take the child up to the ward.

"Is 'e agoin' to stay in?" queried the wretched mother. "I knew yer 'adent the 'art to send 'm out on a night like this."

The doctor rang his bell sharply for the next patient, while he answered in a tone lost on the woman.

"He will probably 'go out' in the night as it is—you had better call to inquire."

Wonderful to relate she did call to inquire for the child many times before the end came, and I have reason to believe that the sight of her baby being properly cared for touched her heart as nothing else had power to do.

In the ward Dolliwag improved wonderfully in appearance. He was washed and dressed in soft clean linen, and tucked cosily into a cot. But clothes sat oddly on him, for he always looked as if he were not a baby at all but a little old man, shrunk and shrivelled down to the size of one. He seemed to find it very comfortable lying there with gentle hands tending him, and bright faces beaming down at him, for he never cried, though he must have suffered a great deal of pain, and he cultivated a smile. That smile became famous; even the head physician noting it one day marvelled at it. It seemed to radiate from Heaven and went straight to our hearts—it even brought tears to the eyes of his hardened mother when she saw it. It was a sad smile, for though it lighted up the baby's face, curving his tight little mouth into a bow and rounding his hollow cheeks into dimpled sweetness, it never extinguished the depths of misery rooted in his big black eyes. But withal it was an exulting smile, for it showed such victory over sin and suffering and that Heaven could make perfect even his imperfect life.

The out-patients had been treated and sent back to their respective homes very early one evening and I hurried up to the ward to help, knowing the nurses would be very busy with a new case which had just been sent up.

I was sent to tend Dolly. When I came to his side his head was turned away but his eyes were wide open as usual, and his tiny irresponsible fingers were clutching the air. Taking them in one of my hands and putting the other under his shoulders I lifted him gently round. As his face turned to mine it broke into one of his most glorious smiles which flickered there awhile then melted gradually away. Then closing his piteous eyes and giving a deep sigh the unloved baby found Infinite Love at last.

### Eight Months at the Imperial Yeomanry Hospital, Deelfontein.

By an Army Reserve Sister.

Now that the war has dragged its weary length along for eighteen months, it is hard to recall the intense enthusiasm and longing with which every other person was possessed "to go out and do something" which characterised proceedings at the outset. Happy the woman at that time who could say, "I am a qualified nurse," though even then it was not easy to get sent out, as the War Office had then, it may be fairly said without exaggeration, the pick of the flower of the nursing world. The first step towards nursing the troops in South Africa was (and is still, one presumes) to join the Army Nursing Reserve, and next, having satisfied the electing authorities at the War Office, to wait as patiently as was possible for the welcome letter or telegram ordering an early date for embarkation. It was my fate to be accepted for service with the Imperial Yeomanry Base Hospital, Deelfontein, and after great preparations and the important purchase of a kit suitable for South Africa the forty selected nursing sisters with the lady superintendent, Miss Mary Fisher, embarked on February 17th, 1900, in the s.s. *Guelph* en route for Cape Town. The voyage out was slow and not too comfortable, but it was with something of the thrill of a new, yet expected, event that we woke on March 16th, 1900, to find ourselves at anchor in the famous Table Bay. We landed the following day, and, amid the cheers of the troops who had come out with us, entrained at 9 P.M. and started full of joyous expectancy for our future sphere of work. The forty sisters were distributed in parties of six in each carriage, and we slept, more or less, on shelves that pull down from the carriage sides. In spite of much dust, we all enjoyed the picnicky life of the journey up to the Karoo and the scenery. At many of the stations kindly English and Colonial folk met our train, and brought us baskets of grapes and figs, lemonade and milk. At one place a late dinner was awaiting us, about 11 P.M., and we were chivalrously waited on by young volunteers, whose assiduity and kindness made up for their want of practice in the art of waiting.

### Our First Afternoon.

As the white tents of the Imperial Yeomanry Hospital came in sight, there was not a vacant window in the train, and a warm English welcome was accorded us. Our first afternoon in the bright South African sunshine, and in the light, exhilarating air of the Karoo, was very memorable, and had all the essence of a "halcyon day." All too soon was the camp to be filled from end to end with the sick and suffering, the enteric huts to overflowing, while one of our welcoming hosts was himself to be a victim, and among the first to lie in the quiet cemetery beyond the camp. We had not been in our new quarters more than a day or so when we experienced our first South African dust-storm, a violent blizzard of dust penetrating everywhere.

### The Health of the Sisters.

It may be interesting to mention at this point the strange attacks of gastro-enteritis to which with scarcely an exception the whole staff succumbed a day or two after arriving on the veldt. Abdominal pain and griping, severe diarrhœa, and occasional vomiting were the principal symptoms. The attack lasted usually two or three days, requiring medical treatment (Dover's powder and bismuth) and rest in bed. Recovery was rapid, but complete acclimatisation was not always acquired until after a second or third attack. Two theories were put forward to account for it: the presence of large quantities of magnesium sulphate in the water, and of irritating and septic properties in the fine dust always being blown about more or less, and getting into the food and water. The health of the sisters was, on the whole, remarkably good, though if once lost it was very hard to regain, while suppurating fingers and sore hands and feet were most intractable and chronic. From personal observation among ourselves and the soldiers, people never felt so well and fit before in their lives if the climate suited them, nor so wretched and unable to pick up again if once below par. The dryness of the air and the height above the sea affected us in different ways, chiefly in producing a certain nervous irritability, falling out and turning grey of the hair, and painful deep cracks on the hands and fingers. Apart from these slight disagreeables, one felt very bright, active, and strong, had an excellent appetite, especially for all sweet things, which everyone indulged in to a large extent, chocolate and sweets of all kinds being always on hand. It was said that eating sweets prevented the craving for alcohol, and as the latter vice is very prevalent in South Africa we liked to think there was some truth in the theory.

### A Routine Day.

Having got our huts and tents ready, and the patients installed in them, it may be of interest to describe a routine day as spent in a hospital on the veldt. Breakfast for the nursing sisters was at 7 A.M., and a good meal of tinned bacon, tinned milk, tinned butter and tea, with bread, all excellent of their kind, was partaken of. At 7.30 a shortened form of Morning Prayer was held in the chapel by the chaplain. The time till 8.30 was employed in putting our rooms or tents tidy, making beds and dusting, or taking a short walk on the veldt in the exquisite early morning sunshine. The wards were all more or less (generally less) ready for the day sisters' arrival on the scene at 8.30, when the first doses of medicine were given, the temperatures were taken all round and charted, the dressings prepared, and all made ready for the medical officers' visits at 9.30. Many of the wards were divided among two or three physicians and surgeons, and contained from 20 to 32 patients, the tents holding 8 to 10 each. The sister and ward orderly accompanied the doctor on his round, the orderly being expected to fetch and carry, and wait generally on the doctor and sister. Among the duties devolving on the sister was the making of jellies for the specially ill patients, the distribution and custody of all the stimulants, and the feeding and care of the helpless cases, besides fomentations, poultices, blisters, small dressings, and splint-padding.

At 12 A.M. two convalescent patients and an orderly went off to fetch the dinner rations for the whole ward, and returned with a huge supply of some nameless stew or occasionally some roast meat. Contrary to the practice in civilian hospitals, the sister has nothing to do with the diets of the convalescents; and as the food was "dished out" by a patient or an orderly, one or two patients would be sometimes entirely forgotten, and there was an instant rush to "Sister," who had to find redress for the injured ones somehow or other. Another fashion, strange to the civilian mind, was the arrangement of the courses. All patients "on pudding" in addition to the ordinary "ration" had it at three o'clock in the afternoon instead of its following after the stew in the usual way. Though uncanny to us, the plan seemed to suit "Mr. Atkins," who is a great believer in the established order of things, and saw nothing amiss in it. After the middle-day round of medicines, the whole ward settled down to a determined "siesta," those who were up and about deliberately lying down full length on the outside of their beds, and enjoying themselves immensely with books and smoking. These practices also needed some getting used to on the part of civilian sisters, but it was easier to unbend out there under such altered and peculiar circumstances.

The sisters then retired to their partition, or "bunk," at the end of the ward, and prepared dressings, &c., for the next day. Our dinner was at 2 P.M., when the orderlies returned from their mid-day meal to relieve us. If the ward allowed, *i.e.*, if the patients were well enough to be left to the orderlies, the sisters were expected to go off duty from 2 P.M. till tea time, as in the Army hospitals the men are not accustomed to the constant attendance of the sisters, and prefer to be left for a time to themselves. Tea was generally laid neatly and daintily outside the door of the hut or tent, and made many a little social centre in the camp between 4 and 5 every afternoon. The usual evening routine, as seen in a London hospital, followed after tea, and with an interval for supper the ward was, so far as the sister was concerned, closed at 8.30; but "lights out" was not sounded till 9 P.M. The night sister came on duty at 8.30, and after giving the night report the day sister disappeared, and was generally in bed before 10 P.M., a moonlight stroll and *tête-à-tête* chat with a sister chum being the usual wind-up of the day.

### A World of its Own.

Our camp became in a short time a small world of its own, a feature only noticeable in hospital life, where the community of interests and the necessarily narrow range of ideas rapidly crystallise into a miniature world, with its religious, social and professional sides all duly developed. With regard to the latter at Deelfontein, as everywhere in South Africa, there was a great preponderance of medical over surgical cases, both in numbers and severity. The cases of enteric fever and other medical cases at one time in the hospital were 462, as against 142 surgical, and the latter were not, as a rule, severe cases. Any severe surgical cases we may have had were, with a few exceptions, the ordinary cases of civil practice (hernia, appendicitis, obstruction, gall-stone, &c.), and not due to injuries received in warfare. The surgical cases did remarkably well, both wounds and operations. The bullet and shrapnel shell wounds often came to us in a very septic state after long journeys from the front, but a few days of regular boracic fomentations cleared everything up. The wounds caused by explosive and expansive bullets were very bad, as so much tissue was destroyed, and they took such a long time to heal. A great feature was made of massage, and two "masseurs' were kept constantly at work on limbs wasted and stiffened' after bullet wounds, and did very good work. A distressing, and unfortunately not uncommon, form of very disabling injury was that caused by bullets damaging the big nerve trunks, with resulting paralysis, wasting and intense pain in the affected part. Operations were frequently undertaken for the reunion of the severed nerve, or the freeing of matted ones; but time, rest, and massage were chiefly depended on for complete restoration to use and power. The pain of this injury when the nerve was not severed, but grazed and matted down, was always most severe and wearing, and produced great depression and hypochondria.

### The Medical Cases.

The enteric cases were mostly very severe. During the hot weather the flies tormented them, fastening on them and their feeding cups in black clusters. It is a well-supported theory now that the flies are great agents in the spreading of enteric fever, and it would certainly seem only too likely to be the case. In spite of mosquito curtains, &c., the plague of flies was a very real one. In a letter written on the spot and dated June 18th, 1900, is the following passage:—"The type of enteric here is very severe, with much hyperpyrexia, delirium, and early heart failure, so that few of the cases are, on the whole, protracted. Many inoculated men have died of it." Of the other medical complaints, the commonest and most serious was dysentery, more intractable and disheartening in its worst forms than any case of enteric. Rheumatism was another common ailment, and was often of a very acute type; while there were an innumerable host of lesser ills, aches, pains, and debilitated conditions, due to the strain of the life, the long marches, and the poor food. A longing to get back to England was almost universal among the men, and before the end of September last year they had all had enough of fighting and South Africa, and were heartily sick of the whole thing.

### How the Wards were Worked.

The system of working the wards with orderlies must not pass unnoticed. After the exact discipline of a civil hospital and the absolute control the sister or charge-nurse has over her ward, it was very difficult at first with mostly untrained men to work the wards comfortably or to get things done except by a great expenditure of patience, good temper, and tact. Occasionally even these cardinal virtues were of no avail, and only the unpleasant ultimatum was left of reporting the delinquent to the ward-master or to the final court of appeal, the sergeant-major—a man of might in the regiment. The regular Army sister is expected to do very little in the wards beyond giving the medicines and stimulants, going round with the doctors, and charting the temperatures. She has nothing to do with the distribution of food or bed-making, &c., all work of that kind being done by trained orderlies belonging to the R.A.M. Corps. At first we all tried to work as we had been accustomed to in a civilian hospital, and to bring the untrained orderlies' standard into line with ours, the result under the conditions existing on the spot being a kind of amiable compromise for which the patients seemed very grateful, whatever the shortcomings, of which we alone, perhaps, were conscious. It is not easy for civilian sisters to work with orderlies, and it would be interesting to hear some regular Army sister's views on the subject, now that it is being ventilated and Army reform is in the air.

### Presentations.

Reigate Borough Isolation Hospital.—Miss Amcoats, on leaving this hospital, of which she was matron for some time, has been presented by the staff with a travelling-bag.

The members of the nursing staff in connection with the York Home for Nurses, have presented Dr. Ramsay, who has been the hon. medical officer for more than twenty-five years, with a handsome silver bowl, bearing the inscription, "Presented to Dr. Ramsay by the York Home Nurses, April 23rd, 1901." It had been intended that it should have been publicly presented on that evening at a dance which the nurses are going to have at the Davy Hall, Davygate, York, but owing to Dr. Ramsay's absence from home then the presentation was made earlier.

### Death in Our Ranks.

The friends of the Alexandra Hospital for Children with Hip Disease, Queen Square, will learn with much regret of the death of Miss L. E. Moore, who was for so many years lady superintendent. Miss Moore during her long service had gained the esteem and regard of the authorities, while her love for children had endeared her to all the patients with whom her duties brought her in contact. The funeral, which took place at Halesowen, in Worcestershire, was largely attended by friends, and included a deputation from the hospital. It is a matter of sorrow to all who knew Miss Moore that the illness which ultimately proved fatal was of a long and painful nature, and attended with great suffering. Miss Moore will be much missed by her colleagues, who were warmly attached to her.

### Nursing in Corea.

By a Correspondent in the Capital.

Nursing in Corea is beset with many difficulties which need patience and careful management to overcome. In this short article I will try to deal with the main difficulties and point out how they are met.

### Lack of Financial Support.

The chief obstacle to successful work in Corea is the lack of proper financial support from home for the hospitals. This is probably accounted for by the fact that some people do not know where Corea is, and those who do know only take a languid interest in the country because it is such a long way off. A stranger travelling in Corea about the time of the Chinese New Year might think that the Coreans as a nation were pre-eminently clean, for then they are decked out in spotless white garments made of calicoes of different qualities according to the pocket of the wearer. The material is now largely imported. Unfortunately, beneath this clean exterior is an unwashed skin—happy hunting ground of that universal accompaniment of dirt the "acarus scabiei." In winter there is some excuse for this lack of bodily cleanliness, for the Coreans have no conveniences for washing except, perhaps, a tin hand-basin; and as their rooms are only 8 feet by 8 feet there would hardly be room for a bath, even if they possessed one. Again, water is an expense, for it has all to be brought from wells, and the carriers have to be paid; few, therefore, can afford more water than is required for their food and washing of clothes. In summer, when river or sea-bathing is obtainable, there is no excuse for this uncleanliness of the people.

### Fighting Dirt.

Dirt is, therefore, the first enemy to be fought, and the routine treatment of patients needing admission is a bath, clean clothes, a worm powder, and a purge. Even this is often not all that is required, for on examining the patient's hair either "pediculi capitis" or their eggs are almost certain to be found, especially when the patient is a boy, for the boys in Corea have large quantities of coarse black hair plaited into a thick queue, using the whole of their hair for this purpose; unlike the Chinese, who shave the front half of their head and only use the hair of the crown for the queue. A patient admitted for a tumour or any skin disease will come in with the diseased part plastered over with a thick black mess consisting partly of tar, which they call "ko yak" or ointment medicine; this all has to be removed before an examination of the part can be made. In summer fresh castor oil plant leaves may be found covering a large ulcer of the back, leg, or some other part of the body. An extensive burn of the arm or other part is shown with the burnt part covered with native ink. Another common application, supposed to be specially good for bone fracture or disease, is "san kol" which is the native word for copper ore. This is found in a cave by the roadside on the way to the ancient capital of Corea, Song Do; it is extracted from the soft rock in the form of small cubes which are seen as shining flakes in the walls of the cave which has been cut out.

### Corean Fever.

In the spring patients are admitted suffering from a fever which seems to be peculiar to Corea and is called by the natives "Yem-pyeng"—it is really a form of relapsing fever. The peculiar characteristics of this fever are bleeding from the nose, and patients suffering from it have a disposition to wander about the compound if they get the chance. These cases need constant watching as they are very apt to get pneumonia from exposure.

### The Only Hospital.

At present there is only one foreign-built hospital in Corea, that of the Church of England Mission at Chemulpo. The Presbyterians will soon have a good foreign-built hospital in the capital, which will be a great boon, for hitherto the work in the capital has been undertaken in Corean-built hospitals, the disadvantages of which are serious. However careful one is, it is next to impossible in summer to avoid periodical invasions of bugs and other insects, which, if once they obtain an entrance, are like the poor, always with us, for they can hide themselves in the crevices of the beams supporting the roof of the ward, and though themselves driven out by sulphur fumes, their eggs are absolutely inaccessible. Sometimes a case of typhus fever is admitted into the general ward, as, owing to the dark tint of the Corean's skin, unless the rash is abundant it is difficult to diagnose. It is not necessary to say much about how the difficulty of dirt is met and overcome. The treatment is the same all over the world—soap and water, a nail-brush, and ether for the grease. Soap has to be used sparingly for economy's sake. The same rule applies to the use of ether. A patient being thoroughly renewed as to his skin and hospital clothes, the right size selected and applied, his own clothes, even if only rags, are collected and put away, labelled ready for him to use when leaving the hospital, for finances will not allow of any gifts in the way of clothing to patients leaving. After attending to a patient's exterior in this manner, he is given a purge at night, followed by a worm powder in the morning. The patient, if a surgical case, is then considered ready for operation. The process of cleansing the wound, if one is present, or surface of a tumour, is sometimes extremely difficult owing to the "ko yak" above mentioned which has been applied. Of all the sticky and nasty messes which Coreans apply this is one of the worst. Most difficult to dissolve, the process of scraping it off bit by bit has to be resorted to before a clean surface can be obtained.

### Heart-breaking Work.

Nursing in a Corean house is heart-breaking work. There are two kinds of houses available—the ordinary house with rooms 8 feet by 8 feet, with a floor composed of mud, supporting flat stones covered with a smooth layer of mud, and over this a layer of oil-paper. The warming of these rooms is by a wood fire which radiates heat through the floor from underneath, a flue running in a zig-zag under the floor and coming out usually at the opposite side of the house, the smoke passing straight out into the open air; or by means of a chimney, if one is added. The natives lie on the floor in these rooms, beds being out of the question in such a small space; and attending to patients lying on the floor is not only heart- but back-breaking work. Besides this, the space between the oil-paper and the mud floor is a happy hunting-ground for all kinds of insects, as is likewise the space between the wall-paper and the wall, which is also of mud.

### Nursing the Richer Classes.

The other houses used for nursing are the houses which the richer classes in Corea use in summer, and have a wooden floor laid in squares. The boards seldom fit well, leaving large spaces through which the ground can be seen. The roof is supported by huge beams, which cross the room. There is no ceiling, but the intervals of the beams are lined with white paper, the windows are of paper, with an outer and inner part, the roof is tiled, but the walls are of mud. These houses make fairly good wards when a new floor is laid, brick substituted for the mud walls and new windows of glass inserted; then, with a stove for warming in winter they are comfortable enough, but the beams have big cracks, which afford good hiding-places for insects of all kinds; cobwebs, too, soon collect unless the roof is constantly seen to. Everything, therefore, except the patients—for Coreans, especially the men, are very amenable to treatment—is against those who undertake nursing in Corea. The houses are difficult to fix up for patients, and when arranged difficult to keep clean; there is an absence of those conveniences always at hand in home hospitals, much trouble in getting patients clean, and when clean, in keeping them so. In contrast to these drawbacks must be put the evident appreciation of good nursing on the part of the natives.

### Lectures to Head Sisters.

By Miss E. Margaret Fox, Matron of Tottenham Hospital.

### LECTURE I.—A HEAD SISTER'S DUTIES TOWARDS HER PROBATIONERS.

(*Concluded.*)

Love of popularity is another pitfall for the unwary. Of course it is very nice to know you are the most popular sister in the hospital, that all the probationers long to come to your ward, and shed tears when they leave it. A really good, just, kind, capable sister generally is popular without trying to be so, but beware of purchasing a cheap reputation by slurring over faults, allowing those under you to do as they like, or by doing an unfair share of the work yourself, and not exacting a fair amount from them.

Above all, do not strive to become popular by depreciating the head sister of another ward. Do not listen to probationers' stories of the unfair way Sister So-and-so treated her. Do not discuss one another with them. And if one of them has received just blame which, as likely as not, she resents, and wishes to make you the recipient of her confidence do not pet and console her as though she had undergone some martyrdom. Few things do so much to spoil the discipline of a hospital. Kindness and sympathy must not degenerate into foolish sentimentalism. Every nurse who enters a hospital should try to remember that she is there to learn to nurse sick people and not her own wounded feelings, and that she should try not to let anything interfere with the main object of her training. Point out to your probationers that it all comes in the day's work, and that even should they get blamed unjustly sometimes, it will not harm them in the end; for good, steady, honest work is sure to be appreciated in due time.

There is another temptation that besets ward sisters, and even otherwise good ones often give way to it. They will take any amount of pains to teach and train their own regular probationers, but if an extra nurse is sent into their ward for a short time, they will not take the trouble to teach her anything, because she will not be staying long with them! This is short-sighted policy, if nothing worse, for you will lose nothing by teaching others what you know, and in a sudden emergency the usefulness of your helpers will be largely due to the conscientious thoroughness of your previous instructions.

So far, we have chiefly considered your duties towards junior probationers. As regards those who are senior, your mental attitude towards them should be to get them ready as soon as possible to take your place. You have to leave them in charge often, when you are off duty and when you have a day off, and it means leaving the ward with a much lighter heart if you feel someone is in charge who will carry on the work as you like it carried on—someone who will not upset all your arrangements, make your cupboards untidy, and allow your patients to get bed-sores. It will depend very much onl your previous training if she will do things in the way you like. Show her how to write the report for the night nurse, how to fill in the various daily forms used in the ward. Let her help you with all the dressings, go round with you when the surgeons or physicians are making their visits, prepare patients for operation, and see anything of interest that may be going on. When you leave her in charge always tell her exactly what you expect to have done before you come back. Do not leave her with a vague order of "getting forward as far as possible," or you will probably feel dissatisfied on your return, because she has not done as much as you think she ought, and she will feel disappointed that she has not succeeded in pleasing you. Let her give the medicines sometimes, instead of you. Give her one or two bad cases to be responsible for. Do not of course, put an unfair share of your work upon her, but let her have the opportunity of doing every part of it, so that she will be able to stand alone in her turn. But take care she remembers you are head of the ward, so that she must not act on her own responsibility, but must refer to your judgment, which should be final on such matters as lie within your province.

With regard to night probationers, some head sisters are rather inclined to be more slack about them than they should be. They do not insist sufficiently on having their orders carried out, and are too ready to accept the excuse of there being "no time" to do so and so. It is obviously unfair to be stricter with those on day duty than on night duty, and yet it is often done. Many night probationers on returning to day duty, are complained of as being slack and self-sufficient, which certainly points to a lack of discipline, chiefly on the part of the night superintendent, but also on the part of the sisters of their respective wards, who are either not explicit enough in their verbal and written orders, or else not particular enough in seeing them obeyed.

It is the night sister's special duty to see that the orders for each ward are clearly given and faithfully carried out, and her responsibilities are almost greater towards her probationers than those of the day sister. She can, if she likes, see more of them, as they all go off duty at the same time, and can help and teach them a great deal if she will only take the trouble. She should be just as strict in exacting punctuality from them at meals, and in coming on and going off duty, and she also should be ever on her guard against listening to gossip and complaints about the various ward sisters. There is more temptation in this direction for a night sister than a day sister, owing to the loneliness of her position, and the greater opportunities she has of seeing and talking to those under her when off duty. She will not find it desirable to form special friendships among her probationers, and while seeking to be kind and friendly to them all should avoid going out with any special one or "making favourites" of any. So surely as she does this she will weaken her authority and lessen her influence for good.

Night and day sisters alike can help their probationers greatly with their lectures. They should seek to arrange the work so that they can easily get off in time to attend them, and if, when the ward is light or there is a spare half hour, they will take the trouble to go over their notes with them they will find a certain benefit to themselves in brushing up half-forgotten facts, and to them in awakening and sustaining their interest in the theory of their profession.

In conclusion, then, we may briefly sum up a head sister's duties towards her probationers as follows: She should treat them kindly, considerately, and justly, giving new-comers a welcome and gradually initiating them into their duties. She must see that the work is done well and punctually and that they learn, not only the routine duties of the hospital, but also the special things belonging to each ward. She should exercise supervision over their health, appearance, and manners, be thorough in what she teaches and firm in requiring what is right. She must do her best to help her probationers to acquire those habits of neatness, order, observation, and quickness that help to make a good nurse. She must avoid favouritism, laxity of discipline and harshness, and strive to give an accurate report of their work and conduct. She should not blame them for what they cannot help nor for what she has neglected to explain to them. Above all she should try herself to be what she would have them imitate if she is really in earnest about becoming a good ward sister.

### Across the Seas.

### NURSING AT WINNIPEG GENERAL HOSPITAL.

By a Graduate of 1899.

When I was a girl of nine years old I well remember seeing in an illustrated paper a small picture of what I considered to be a very fascinating person; the little sketch was that of a hospital nurse in the usual immaculate uniform; and I there and then determined that when I grew up I would if possible become a reproduction of this charming ideal. When I grew up an opportunity occurred for me to enter the training school attached to the General Hospital at Winnipeg, Manitoba. I did not let the chance escape, but became a probationer, in the spring of 1896—looking forward to a three years' course of hospital training—a life, about which I also knew absolutely nothing, and of course, I had a very vague idea of the responsibilities, pleasures and trials which fall to the lot of any nurse. A month after sending in my application and photograph (my home was 1,500 miles from Winnipeg) I received a telegram telling me that there was a vacancy in the hospital, and that it would be kept open for me for a week. I started on my three days' journey two days later, and after spending a day and a night in Winnipeg, with friends, I entered the hospital at 2 p.m. on an April afternoon.

### Hours of Duty.

I found that the hours on duty were from 7 a.m. to 7 p.m., with one hour off in the middle of the day, one afternoon free during the week, and part of Sunday, either from 10 a.m. to 2 p.m. or from 2 p.m. for the rest of the day. We rose at 6 o'clock and breakfasted at 6.45. Dinner was at 11.30 and tea at 5.30; besides these meals a cup of tea was served at 10 in the morning and at 2 in the afternoon, which was always most welcome and much appreciated. The doors of the nurses' home were closed at 10 p.m., and lights were put out at 10.30. Anyone coming in later than 10 o'clock had to enter by the hospital door, and write her name in the book which was kept for that purpose. The ward was in charge of either senior nurses in their third year, or graduates, the head nurse having two, three, or four pupil nurses and a probationer under her, according to the number of patients in the ward.

### As Probationer.

I began my training in the men's medical ward, my head nurse being a graduate of the Training School, who was then and has always been my ideal of a head nurse. During my first month of probation my time was chiefly occupied in learning the routine of the ward, and the one and only way to dust, sweep, scrub, polish, and generally clean everything contained in that ward, with the exception of washing and scrubbing the floors, which was the duty of the ward-maid. My second month's probation was more interesting, for I learnt how to take a temperature and respiration, to keep daily, two-hour, and special charts, how to prepare poultices and fomentations, the proper method of sponging a patient, and the right way to give medicines and prepare for simple dressings. I do not suppose that one's probation is ever a very happy time in any hospital, but I think I felt more miserable and appeared more hopeless than most probationers, for, added to my awkwardness in doing strange and unaccustomed work in one particular way and no other, I was terribly homesick and lonely amongst so many strangers and such a novel sort of existence. Many were the times when I scrubbed baths and polished brass with soap mixed with tears. My reward came, however, when my head nurse, who was always chary of praise, told me that I polished brass better than any probationer she had ever had, and that she would really miss me when I went to another ward. When she spoke of my going to another ward I hoped it meant that I was to be accepted as a pupil nurse, but I was not at all sure, and it was a great relief to me when, one day, the lady superintendent stopped in her rounds of the ward and told me that I might apply for my uniform to the housekeeper, and appear in it the first Sunday after my two months' probation was finished.

### As Pupil Nurse.

My uniform was made and ready to be worn by the appointed Sunday, and I got up at least an hour earlier to give myself plenty of time to put on and arrange becomingly the large white apron and bib, the strings of which took a novice at least ten minutes to tie satisfactorily, while the starched linen cap required much more "fixing" than the ordinary muslin one of the humble probationer of yesterday. I slipped down to breakfast earlier than usual, as I almost dreaded the congratulations and comments which I knew fell to the lot of every probationer when she made her *début* as a pupil nurse. My head nurse smiled approvingly at me when she came down, and most of the other girls congratulated me or commented on my appearance in some way, and the lady superintendent, taking me into the ward kitchen, kissed me and welcomed me most kindly to her "flock" of nurses. This all had the effect of making me feel more at home, and that I was really beginning to belong to the staff of the institution and to be of some slight use in the working of it. It took me at least a week to get used to my uniform. In fact, coming down a corridor one day, I saw coming towards me a tall and to me an unknown nurse; it was a minute or two before I realised that it was my own reflection in a glass door. My greatest sorrow at that time, and it was to me a very real one, was that I could not learn to take a pulse with any exactitude. If it was a man's pulse, strong and even, I was all right; but when it came to that of some feeble woman or child, I was completely at a loss; and I remember sitting on my bed one night and almost weeping at my failure, as I thought that I should never be able to make a smart nurse, for it seemed to me that to be able to count a pulse correctly was the pinnacle upon which a nurse's reputation stood or fell.

### As Third Nurse.

I had then been in uniform for a month, and had been promoted to third nurse in the ward, when, one morning, I was asked to go to Section D in the Isolated Block, the nurse in that section being sick and having to go off duty at once. I said I was sure I couldn't manage a ward, however small, alone, as I did not know how to do so. But the head nurse in my ward replied, "Oh, yes, you can; I've been teaching you to do it all the three months you've been with me. You can go to the head nurse there for advice at any time, though she will never come into your ward except in an emergency. I am sorry to have you leave me, for we'll miss you in the ward. But please don't ever forget that you were my probationer, and do me credit wherever you go in the hospital." Then she dismissed me to go to my room to pack up enough clothes for a month's stay in the Isolated Block. How I longed for the time when I should be training probationers and telling them to do me credit, instead of being a mere junior, at everyone's beck and call, and trembling at the mere thought of any responsibility. In half an hour the Lady Superintendent came to my room and told me that if I was ready she would take me to the Isolated Wards. I think she saw how nervous I was about this change, for on the way over she explained to me that the ward I was to have charge of was used for measles, and that none of the cases at that time were very serious, though the wards were full, and she also told me that the head nurse would always be at hand to give me advice when I needed it, though she expected me to be capable of running the ward fairly well by myself. After introducing me to the head nurse she left, and I was escorted upstairs to Section D, where the night nurse, who had been obliged to stay on duty till I relieved her, took me round to the wards and explained the work to me.

(*To be continued.*)

### Hints on the Practice of Massage.

By A Trained Masseuse.

There are a great many people who learn massage at the various schools for the teaching of the profession who find it in many ways, in the starting of a practice, a much more difficult thing than it appeared to be in the training school. If possible after obtaining a certificate it is best to work under a good masseuse; but as this often cannot be done it may be of use to some masseuses to tell them of some things which I have learnt by my own practice. It is very necessary to have confidence in oneself; this can be gained by experience only, but it will be quickly acquired if one is successful in the treatment of patients. Confidence from the patient is equally important, but as a rule the one follows the other.

### The Time for Application.

The sole object should be the cure of the patient, and no means to this end must be disregarded. For instance, to apply massage at the time a patient wishes it will in all probability make a difference in hastening the cure of the patient, and so if it is an inconvenience to oneself it is fully repaid by more quickly gaining one's object; though with regard to time the masseuse must watch each patient carefully to see what time seems to suit them best. It is a treatment that has very different effects on different people—with some a stimulating and with others a soothing effect even when given in the same way; so if the evening does not seem to suit a patient, one must try the morning and *vice versâ*. If there is dislike on either side, the patient disliking the masseuse, or the masseuse the patient, the case ought to be given up at once, for little good will be done. It may not mean that the massage does not suit the patient, as with another masseuse—be the method similar—it may have quite a different effect. But a masseuse soon learns to adapt herself to the patient, and where she can do so it is seldom necessary to change.

### A Love of the Work Necessary.

No one ought to go in for the profession who does not thoroughly enjoy the work, for it is impossible to treat a patient properly if one does not care for the practice of massage. Each one needs so much care and thought, and—as in anything connected with human beings—there is a good deal of responsibility. Undoubtedly, the attitude of the masseuse's mind affects the patient. In this way, and also audibly, one can use suggestion in trying to relieve pain, &c. I have often said to a patient while applying massage to a painful part, "The pain is going" . . . "It is getting better" . . . "Now you hardly feel it," being confident that what I say is really taking place. It is also quite possible to alleviate pain by simply laying one's hands on the painful part and concentrating one's mind on removing the pain. This power will greatly increase with practice. It is very seldom that either massage or movements need be painful to the patient. Should it be impossible at first to move a joint without causing pain a few minutes of even rubbing and kneading will often obviate this. It will also in many cases produce temporary anæsthesia of the skin.

### Avoid Hurry.

Punctuality in attending patients is essential. If the masseuse is always punctual at her patient's house she will generally find that her patient is ready for her. As a rule it is advisable, when it is possible to arrange one's patients far enough apart, to be able to give a little overtime if needful, so that the patient should have no sense of hurry, however full is the masseuse's day. A masseuse can only judge for herself how many patients she can take in a day, but she will soon find that it is not fair to them to take more than she can well manage without over-fatigue, as when very tired she cannot do her patients much good. It is advisable not to attend more than two patients together without taking some sort of refreshment or rest.

### The Choice of Lubricants.

With regard to lubricants, every masseuse has her pet ointments, powders, &c., and this is certainly a detail, as it is the massage itself that does the good and not the lubricants. If a patient has any special choice as to what should be used, unless I have any real objection to it, I generally use it. Where the skin is very dry and needs nourishing an ointment is preferable to a powder, and there are some embrocations, &c., that seem to help to alleviate pain though the directions on most of them—"to be rubbed well in night and morning"—plus the amount of belief a patient has in them, are more often the secret of their virtue than their ingredients. Before applying massage careful diagnosis is needed. Except by very experienced masseuses, and then only seldom, it ought not to be undertaken without the advice of a doctor.

### Discrimination Needful.

Never use a movement that is objectionable to a patient. It is not likely to do any good, and one can often substitute another that will have much the same effect, or after a time, when the patient is quite used to the treatment, she may not mind any movements which one wishes to give. I know of some masseuses who use all the movements quite indiscriminately with regard to the complaint. A patient came to me some time ago suffering from some affection of the spine, telling me she had been advised to have massage, but that it was painful and irritating to her, and seemed to do her no good. After going thoroughly into the case I questioned her as to what sort of masssge she had had, and I found that vibration movements had been given with a good deal of force when only efflurage (at any rate at the beginning of the treatment) should have been used. Never touch a patient gingerly. Some cases need very gentle handling, but that is quite a different thing, and to such, evenness of pressure makes all the difference. In cases of gout very light handling causes a great deal of pain where firm and even friction is soothing. To a certain extent one must be guided by the patient's feelings what amount of pressure to use. Do not, either, touch a patient with cold hands—they can always be quickly warmed in hot water. The masseuse should be careful to keep her hands soft.

### The Use of Electricity.

I have always found that electricity depends more on the patients' temperament, &c., than the ailment from which they are suffering, and upon the liking that the patient has for that form of treatment. In similar complaints I have found it do wonders towards recovery in one case, and only produced irritation and sometimes neuralgia in another. Where applying it to the face or head it is better to do so through one's own hands, attaching the reophore with a wristlet and letting the patient hold (or be laid on or in some way attached to) the other pole. It is always better to give too weak a current than too strong. While a mild current strengthens a weak nerve or muscle, one that is too strong will weaken it still more. It depends rather on the skin of the patient (whether it is thick or thin, moist or dry) than anything else, how much the electricity is felt. The masseuse should always test the strength of the battery herself before applying it. Some months in a hospital will be of great use to a masseuse. She will become acquainted with various diseases and their treatment; also with bandaging, and this is a thing that all masseuses ought to learn, as it will often be necessary to bandage a limb or joint.

THE QUESTION OF FEES.

With regard to fees, when the masseuse is independent of nursing homes and institutions she can of course charge what she likes, and this ought to be according to what she thinks the patient can afford. The full fee would often exclude the treatment from a patient to whom it would do a great deal of good, and the masseuse will soon forget that from some of her patients she gets only half fees when she sees the successful result of her work. The length of the course of massage ought also to be considered, and reduction made if the treatment is to be continued for a long time. I think it is generally better to include travelling expenses, lubricants, &c. in the fee named to the patient.

A PROFESSIONAL DRESS SUITABLE.

Uniform is also merely a matter of opinion and choice. To my mind a professional dress is the most suitable, and it has many conveniences—one of them being that it forms a protection to the masseuse when out very late as is often necessary in treating patients for insomnia. There is much objection nowadays to the uniform on account of people who are not nurses wearing it; but I can see no reason in that why those who have a right to the dress should not wear it. Why should we hand over our uniform to those who have no right to it, and—as so many nurses now do—say in effect "Very well, if you want to wear it you can and I won't." There are, of course, other reasons why many masseuses prefer private dress—*i.e.* when they are cycling—and as long as they are suitably and loosely dressed it really is of little consequence.

CHRONIC CASES.

Every masseuse is sure to have some chronic cases—patients for whom massage or any other treatment does not cure, only alleviate, or where it merely keeps the muscles from wasting, as in some forms of paralysis, or arrests the advance of a disease. They are often discouraging: day after day the patient seems the same with little or no improvement and at times, perhaps, it is only natural that one is apt to take less interest in them than in other cases. But one should never lose sight of the possibility of cure, and to these patients, to whom the hopelessness they often have of their case intensifies their sufferings, one must give one's very best and try to make the time of massage as bright as possible to them. I have always felt far more for such than for any other patients. People get used to seeing them suffer and they often have little sympathy given them, but they can never get used to it themselves, however bravely they bear pain and weakness. It is certainly better not to talk to patients about other cases, even where no name is mentioned, unless it is likely to give them more hope for their own recovery.

THE TEACHING AND PRACTICE OF MASSAGE.

The art of massage and applying electricity is now thoroughly taught in many schools. It is best to occasionally visit the school where one has been trained as, like everything else, massage advances, and there are often new things to be learned. I always make a point of doing so every year, and have found it very useful—each time gaining some fresh idea or information. A masseuse cannot but find her profession intensely interesting, if she really cares about the work to begin with, and gives her whole mind to it, and this interest will grow with every fresh successful case. In time she will find that there are very few pleasures that can compare with those she gains in seeing her patients recover, and at such times she feels that her life is well worth living, for what most women chiefly desire is to be of use in the world.

### Nursing at a Base Hospital in Natal.

CHAT WITH AN ARMY RESERVE SISTER.

NURSING sisters from South Africa seem to have been arriving in England in small crowds during the last few days: the *City of Vienna* brought three, Sisters A. and F. Price and M. Galloway; then came the s.s. *Dunera*, on Tuesday in Easter week, with Sisters Atkins, Moxon, Sands, Millar, E. Andrew, and Leng. On the same date, too, arrived the *Pinemore*, bringing Sisters E. Croft and A. Myring and lastly, on Thursday, the s.s. *Catalonia* came in with the following on board—Sisters Squire, J. McLeish, M. Leonard, and J. Tuigt (colonial).

With Sister Myring (A.S.N.R.) I have just had a chat, and she has told me something about the voyage home, as well as about her experiences in the base hospital at Howick, Natal. Her life in South Africa has been neither a long nor an eventful one: she has not been to the front, nor has she been fortunate—or unfortunate—enough to be shut up in a besieged city. But she has, nevertheless, done good work at Howick base hospital, and has, I judged from what she told me, been able to adapt herself with ease to working under Army regulations, which, as everyone knows, are very different from those of a civilian hospital.

FROM PRIVATE WORK TO ARMY NURSING.

"I suppose," I said, "you had a few weeks' preliminary training in one of the military hospitals at home before going out?"

"No," Miss Myring replied; "I was sent directly to Howick. But I think it is not difficult to fit into the new conditions in which one finds oneself; and though I do not think it is an ideal system, I was quite able to adapt myself to it. It seemed at first as if, although the sister was always appealed to in everything, a great deal was taken off my shoulders. The responsibility of keeping the ward clean, for example, rests with the sergeant, while the sister's time is occupied with the actual nursing, giving stimulants and medicines, taking temperatures, and so forth. You have to be very firm with the patients, for they take advantage of you if they can."

"Do you like the system of orderlies?"

"Orderlies are very useful indeed for cleaning and that kind of work, but when it comes to nursing I don't think the average orderly is sufficiently educated to do it well. I know they are very necessary, but I am of opinion that a sister with a probationer under her could do very much more. Of course that is what I have been accustomed to."

"You were trained in a London hospital, were you not?"

"At St. George's Infirmary. Then I went to Grantham and Nottingham Hospitals, where I was sister. I was private nursing just before I was sent out to South Africa."

"How long ago was that?"

"I went out last June."

"And you have been fairly busy ever since?"

NURSING ENTERIC CASES.

"Yes; we had a great many enteric cases, especially from Harrismith. The hospital was originally built for 1,000 patients, but only the tin huts are now in use, the tents being closed, and the number is reduced to 500. There is electric light in the wards, and the theatre is a very good one. We also have the X rays in use."

"How many sisters are there?"

"Eighteen now. There were twenty. Two came home in the *Avoca*; one joined Baden-Powell's staff. There is a ward for Boer female patients, in which enteric has been the chief illness, as indeed it has been throughout."

"Had the hospital been long opened when you arrived?"

"Oh, no. I was there for the opening. Sister Kerr is the sister superintendent. The life is a very free one compared with life at home. There is no trouble about dress, for example; I like it very much for that. And Howick is a very pretty place. It is near the falls," and Miss Myring crossed the room to fetch a number of photographs, one of which showed the river falling down a sheer precipice of rock into a foaming pool.

"And here is one of my ward," she continued, "and this again is the officers' ward; but it is different now: they all have cubicles. Here is a group of wounded officers: one is on crutches on account of rheumatism, and another has a gunshot wound. They have all returned home."

THE "SISTERS' AMBULANCE."

"This," said Miss Myring, "is a photograph of the Sisters' Ambulance. It was commandeered for them, and was brought to Howick after the siege of Ladysmith, when the P.M.O. came down, bringing his nurses with him."

"They travelled in the 'Ambulance'?"

"Oh no, it was too far for that. It was sent by train, and I suppose the mules which are drawing it came by train too." The carriage, which was standing outside the hospital, was in charge of a swarthy-looking driver, and the words "The Sisters' Brougham" were written across the corner of the photograph.

"We do our shopping in Maritzburg," said Miss Myring. "Here is a snapshot of the scene there when General Buller arrived." The scene consisted of hats and umbrellas—for there was a glaring tropical sun—and mottoes of welcome appeared on the public buildings.

LIKE AN ENGLISH VILLAGE.

"General Buller also visited Howick Hospital, and seemed pleased with all he saw. Howick is rather like an English village. There are not many trees, but strange fruits, of which I cannot remember the names, grow there, and the air is delightful. It is so healthy that I have very much benefited since I went out. The fever season, too, is over now, and people are very well there."

"Are there many residents?"

"Not very many; those who live there are, however, exceedingly kind. We have had many presents to the hospital of fruit, &c."

A PLEASANT VOYAGE HOME.

"Speaking of presents, did you receive any from the various funds for the men during the voyage?"

"No; I believe the funds have run out. But we had a very comfortable and pleasant voyage, though it was uneventful. The heat of the tropics was great, and we were very glad of the electric fan. Though there were nearly a thousand men on board, there was very little illness, and only four cases went to Netley. One poor fellow had quite lost his reason, and had to be kept under guard night and day. There was one case of pneumonia and about three of enteric. About two hundred men joined us at Capetown, and on account of the plague we were not allowed to go on shore at Las Palmas, where we coaled. The men had frequent medical inspection—about every three days, I believe."

"You were not, however, kept in quarantine at Southampton?"

"No; we had been twenty-one days on the voyage, so there was no necessity."

"Were there any other ladies on board?"

"Besides Sister Croft and myself there was a clergyman's wife. Sister Croft is from Australia: she came from Mooi River, where, I think, she had been about twelve months. There were eight time-expired civil surgeons on board."

"Are you returning to Howick?"

"I got my orders yesterday; I am to sail on Saturday in the *Dunera*."

### Nurse Ward's Packing.

"GOOD-BYE, dear Nurse. I wish you were not going to 'the Front.' Come back safely, and take care of the money."

"Be sure I will. Don't worry about the cash. I have packed it safely, and don't intend the bag to leave my hands."

Nurse Ward stopped abruptly. She had seen a glance pass between two shabbily dressed men on the platform as she and the daughter of her late patient made their way to the train. She did not remark upon them to her companion, but bidding her adieu with many thanks for much kindness received, she chose a carriage in the centre of the train, attracted by its occupants—an old lady with a beaming face and a much younger woman. The small luggage rack was full one side, so she thought she might have their company to London. Standing to watch Mary Gray after their farewell, she saw the two men—a rough-looking couple—apparently leaving the station, and smiled to herself at her fears. The old lady made room for her.

"You seem to regret parting with your friend?"

"I do; it has been a long, yet good, satisfactory case. Generally I dislike an engagement in an isolated country place, but for true hospitality and kindness my stay in Bucks will be remembered with pleasure.

. . . . . . . .

"It's no use our getting in now, the other people get out at Wynham, I saw their trunks labelled to change there. As they leave we get in, and afterwards induce her to give up the bag she means to hold tight on to."

"All right, it's rather risky, and I don't think will pay as well as crib-cracking; but if we leave things until the last and then bolt, we may succeed. I don't half like the job. It's a shame to rob a nurse, and they were good to little Jack at Bart.'s. I don't mind if we don't get the swag."

Messrs. Moore Brothers, burglars and general scoundrels, had not left the station. After a brief consultation they had taken tickets to a market town where the train stopped after a rather long run.

Nurse Ward found her fellow passengers very sociable, and the time passed quickly, and it was with mutual goodwill they parted at Wynham.

. . . . . . . .

"Here you are—this will do." The two men had changed carriages.

Nurse Ward was startled. She had jumped to the conclusion that they had left the station. At first no movement was made, and she continued her search in the timetable to learn when she would reach town, but her senses were on the alert. Seeing the alarm bell quickly and quietly removed and slipped into the burglar's pocket, she tightened her hold upon her bag and stood up, meaning to put her head out of the window and call for help. Her idea had been anticipated. Whilst one of the men blocked the window with his back, the other grasped her wrist with an iron grip to get her hand from her bag.

"Now then, nurse, it's no use showing fight—keep that sort of thing for the Boers. We are in the middle of the train, and the guard won't hear you, however loud you may squeal. You know what we want—give it up I say, give it up." The fellow's tone was brutal.

Suddenly her hold relaxed. He laughed when he found the bag unlocked.

"Very careless on your part, nurse," he said, opening the bag and drawing out its contents—some letters, a roll of lint, a number of reels of plaster and some wooden boxes. The lid of one he removed, then threw them to her with an oath. "Golden ointment indeed!"

At the bottom of the bag, hidden under a woollen wrap, he found a good-sized soft wash-leather bag, tied at the neck with strong silk cord and marked " £5 " in scarlet letters.

Nurse Ward made a snatch at it. " Oh ! take the rest, and welcome ; but leave me this ! " she cried.

" Not exactly ! it's the only thing I want out of the whole lot. This makes pretty music, doesn't it ? " holding the bag above his head and making the contents jingle. " No wonder you made up your mind to keep it."

" Time's nearly up," said the other man. " We close into B——." The nurse made no resistance when the roll of lint was wound over her mouth and her hands held as long as the train remained at rest at the station.

Then bidding her a mocking good afternoon they jumped out as the train moved slowly off, landing on the extreme end of the platform. The ticket collector looked at them.

" I went to sleep, guv'nor, and my pal has more than he can carry."

" So I should say," and thinking that the jump from the train was a drunken freak, he let them pass after receiving their tickets, watching them until they had walked unsteadily down the stairs and into the street.

. . . . . . . . .

" I should like to kill her," said Mr. Moore a short time afterwards, and judging by the expression on his face he meant what he said.

" Would you ? I'm jolly glad the nurse got the best of it," growled his brother.

. . . . . . . . .

Extract from a letter from Nurse Ward to Miss Gray :—

" And now, dear, after giving you all the news of my doings in South Africa, I must explain how my adventures on the railway the day I said good-bye to you in dear old England came to so harmless an end, and how it was I did not lose the £73 I had received from your mother for nursing duties during the ten months of my stay."

I had packed the sovereigns in twelve empty ointment boxes, and to keep them firm filled up the intervening space with carbolised vaseline, and in a spirit of humour had written upon them " Golden Ointment."

The soft leather bag for which I pretended to make a fight contained brass counters made up as coins for Christmas games. They were intended for my brother's children, and would not have been in my bag had my trunk not been full.

The thieves had in their hurry carried off one box containing the odd sovereign, just to show them of what the " Golden Ointment " was composed.

### Ethics of Nursing.

### COURTESY.

When I was a child my father was very fond of quoting to me the words of the wise man, " A gracious woman retaineth honour," and now that I am bordering upon middle life, those words often recur to my mind with renewed force. In these days of rapidity, brusqueness, and lamentable want of manners, I long to illuminate the text, and present it to every girl of the rising generation. For the characteristic of the young woman of the day is not graciousness ; whether because she considers courtesy old-fashioned, and brusqueness more modern and honest, or because she looks upon good manners as insincere, I do not know. The fact remains, that the modern woman, in every class of life goes so far as to cultivate bad manners and ungracious ways.

It is a pity !

This ungracious spirit, this lack of manners, is as noticeable amongst nurses as amongst other women of the community, and in their case I lay some of the blame upon their hospitals. Why should it not be made an essential part of a nurse's training, the training in courtesy ? Without a doubt in those hospitals where the matron is gracious and polite, and where the sisters of the wards follow her example, we find that the nurses also aspire to good manners. Whereas, what can one expect from the nurses in a hospital where the heads look upon ordinary civility as unncessary. For example, a friend of mine entered the office of a large London hospital not long ago, on business. She was not in uniform, but merely a lady wishing to speak to the matron. The assistant matron was sitting in the office, and she never even rose to greet a stranger, and a visitor ! She remained seated. Can bad manners go much further ? And what can you expect from the nurses in that hospital ?

What possible merit can there be in discourtesy from any point of view ? People say, " Oh, those good manners cover insincerity. A person who is so very ultra-courteous to your face is quite different behind your back. It makes you untrue to yourself to be pleasant to everybody, whether you like them or not ? "

May I reply, " Rubbish ! "

Good manners and insincerity are not synonymous terms. A truly courteous person simply puts himself in the place of those to whom he speaks, tries to set them at their ease and make things pleasant for them. True good manners are not rooted in dishonesty but in love of others, and in sympathy and that power of imagination which enables you to put yourself into another's place. And why, in the name of fortune, should you be rude to an unlucky person because he has been unfortunate enough to incur your dislike ? It is rather a case of adding insult to injury.

There never can be any reason for being discourteous anywhere. Who was it who said that a " real lady " had the same manners for her drawing-room and her kitchen. Equally a nurse should have the same gracious manners in her ward or in a private house.

Nothing is more trying to the casual visitor in a hospital than to be met at the door of a ward by a forbidding-looking individual and asked freezingly, " What is your business ? " A pleasant smile, a courteous word, would set the stranger at his ease ; and, really, they are no trouble to the nurse.

The opposite extreme of bouncing, brusque familiarity affected by some nurses is quite as bad as is frigid rudeness, and this bouncing sort of manner is in the worst possible taste when it is used towards doctors and students. I was observing a nurse the other day at a private case, and, though her manners were on the whole good, she had a little flippant, nonchalant way of addressing the doctor which was wanting in good taste. My own strong feeling is that a doctor for whom one is working should be addressed as " Sir," not as " Doctor." Some nurses are idiotic enough to think it is derogatory to use the word " Sir "—that it implies a lowering of their social standing, or some nonsense of that description. Of course it is nothing of the kind. A house surgeon does not lower his social standing because he addresses his visiting surgeon as " Sir." It is the merest matter of etiquette and good taste, and it should be the same with a nurse. No well-trained nurse would address the doctor either by his name or as " Doctor." She would say " Sir." " A gracious woman retaineth honour." Why not be gracious and courteous to all with whom you come in contact—your hospital patients, your fellow nurses, the doctors and students, your private patients ? Life is short enough, and, for many people, hard enough. Cannot we use it to be loving and pleasant and courteous and helpful to others ? Good manners do oil the wheels of life ; they make rough paths so much smoother. We know so little of the worries and troubles of others, possibly brusqueness from us may make another person miserable where a kindly smile and courteous word would cheer him on his way.

" The greater man the greater courtesy," sings the poet ; and some other words come to my mind which I have often longed to hang in the room of every hospital nurse, where they may sink into her mind and be practised in her daily life :

" For manners are not idle, but the fruit
Of loyal nature and of noble mind."

### Echoes from the Outside World.

### AN OPEN LETTER TO A HOSPITAL NURSE.

THE wisdom exhibited by the King in allowing the Duke and Duchess to go on their prearranged tour round the world is already bearing fruit in the gratification which seems to follow some of the announcements made by the Heir-Apparent in reply to addresses. When the Duke of Cornwall landed at Aden he foreshadowed a possibility that at some more distant date he may be able to pay a visit to India, and now at Ceylon he has shown that his desire is not to extend the Empire by bloodshed and war, but by more peaceable methods. "The struggle between nations," he said, in thanking the Chamber of Commerce for its address, "is one, not of arms, but of trade; and it is to Chambers of Commerce, the eyes and ears of our national commercial system, that we turn for help and guidance." As of course the substance of these speeches had been discussed before he left our shores, they are really of importance as representing, not merely the ideas of the Heir-Apparent, but of the King himself. Ceylon, alluded to as the "beautiful island," has an almost historical charm to the Royal pair, for not only is this the second time the Duke has paid it a visit—he was there nineteen years ago as a youth—but twenty-six years ago his father laid the foundation-stone of the great breakwater, which has so largely contributed to the growth and prosperity of Colombo. One of the presents given to the Duke on Saturday had a distinct interest of its own. It was a cluster of King cocoanuts and a jak—a green melon-shaped fruit—both of which were grown on trees planted by the King in 1875 at an old fort about 100 miles from Kandy. The Duke was also informed that the trees which he planted himself in 1882 were flourishing, though they have not yet arrived at the stage of fruit-bearing.

A great many functions took place during the stay of the Royal visitors, but one of the most interesting was at Kandy, where a grand reception was held in the audience hall in honour of the Kandyan chiefs. There was a brilliant assembly, the chiefs being attired in gorgeous costumes, bedizened with gold embroidery, medals, and jewels. All were barefoot, and behind them came the minor headmen, who were naked to the waist, wearing only white comboys from waist to ankles and red or white flat pancake-shaped hats. At the express desire of the Duchess, two Kandyan ladies, though these invariably hold aloof from all public gatherings, were presented to the Duchess. I may add that, though the Duchess is said to have taken with her 300 black dresses, she wore a pearl grey one at Colombo and a white one at Kandy, whilst the Duke donned a white naval uniform on the one occasion and a grey coat on the other. I am so glad to note that commonsense was allowed to rule over even the exigencies of Court mourning.

THE report of the Registrar-General of Births, Deaths, and Marriages is especially interesting reading this year, because the facts which it records are decidedly unexpected. Throughout the whole of the past eighteen months the shadow of war has been over the land, entertainments have been rare, money has been scarce, and many a sad tale of a *fiancé* slain in battle or by the enteric scourge in South Africa has reached our ears. And yet, to the surprise, I should fancy, of most of us, the figures now to hand prove that the marriage rate has been higher than for the past 25 years. The number of marriages registered was 262,334. The proportion of widows who re-marry goes steadily down, but the same difference is not noticed in widowers, probably because, poor men! they find it so difficult to manage a household with servants and children without a woman to assist them that, in despair, they once more take unto themselves a wife. As to births the baby boys did their little best to readjust the preponderance of females in the population, for the number of the male infants born was nearly 18,000 more than that of the females, though I suppose, considering the fearful number of men whose lives have been lost in the Transvaal, the difference will not even prevent the balance of the sexes falling still lower than it has fallen of late years. One little piece of information furnished by the Registrar will please all the admirers of Mr. Walter Long, and must even do something to soften the hearts of his detractors. After gradually dropping during the three previous years from 8 to 6, and then to 2, the deaths in 1899 from hydrophobia came down to nil. And yet some people still refuse to believe in the efficacy of the muzzle!

YOU will remember that I told you about the scheme for establishing a number of public-houses under the auspices of a Public Trust Association. The movement has made so much progress that it has been decided to form in London a central association to which public-house trust companies can apply for assistance and advice. Local committees, with the object of constituting trust companies, have been, or are being, formed for Durham, Essex, Hampshire, Hertfordshire, Northamptonshire, Nottinghamshire, and Surrey, and for Edinburgh, Glasgow, Leeds, London, and Paisley. We shall soon, therefore, see the practical effect of a new departure in which women are not less interested than men. Earl Grey, with his enthusiasm and energy, is a host in himself, and Mr. Oliver Williams, of 71 and 72 King William Street, the secretary and treasurer of the Public House Trust Association, who is not unknown to nurses, is a capable lieutenant.

IF the experiment to be tried by the West Ham Board of Guardians is found to be successful, it is probable that it will be largely copied by other workhouse authorities, and also by those in charge of crèches and homes in the poorer parts of our large towns. The West Ham Guardians find that a large proportion of the babies under their charge do not whilst of very tender years, get the fresh air which is needful for their well-being, because there are not sufficient attendants available to push the great number of perambulators required to take out all the babies in the nursery. Accordingly, they have ordered a specially roomy "omnibus-perambulator to be constructed which will accommodate twelve babies at once, and it is hoped that the vehicle will be sufficiently light to enable one able-bodied person to propel it along. If lightly built, there is no reason why it should weigh more with a dozen wee babies in it than does an ordinary bath chair containing a fat lady or gentleman, whom it is no uncommon occurrence to see at watering places pushed by a longsuffering daughter or nurse. I conclude that these vehicles will not be allowed to frequent the most crowded parts of the town, or a procession of three or four "omnibus-perambulators" would effectually block up the pavement; but in public parks or along country lanes there should be plenty of room for the poor little "Oliver Twists" and their sisters to drink in the fresh air in their carriages, and thrive apace. In poor districts an enterprising person might easily make a small fortune if she purchased one of these latest inventions and undertook for a trifling sum per hour to take the baby off the mother's hands, and also provide it with the necessary exercise. I am anxious to learn what precautions are enforced to prevent an obstreperous baby from damaging its neighbours; or are such trifling details left to Nature and Chance?

### Novelties for Nurses.

By Our Shopping Correspondent.

### MESSRS. E. & R. GARROULD.

At their well-known and always popular shops in Edgware Road Messrs. Garrould have, as usual, a tempting show of novelties for nurses. Among other admirable accessories may be noticed a Russian leather wallet which folds into a very compact shape, while when opened out all the contents are exposed to view in their proper fittings. A very useful variety of the pulse-glass is made with a pencil at one end, the glass being at the other; it has the advantage of being both compact and time-saving, for when the pulse is tested the instrument has only to be reversed for entering on the chart. Gage's "nickel tongue depressor" is another combination instrument, in which tongue depressor and clinical thermometer are combined. The well-known leather pin-cushions are now made in dark blue, as well as scarlet, and in this department are some remarkably inexpensive watches in engraved silver cases, with the Red Cross enamelled in bright red in the centre. Chatelaines, badges, belts, &c., are shown in great variety, and among the latter is a remarkably cheap white washing belt which costs 4½d.; it is made of strong linen-finished cloth, and the sizes in stock are from 20 inches to 29 inches. Nurses are usually too busy to spend much time over the preparation of such necessary things as caps and cap-strings, and they will be glad to know that the "Olivia," though a very good and pretty shape, is quickly made up: this is a great advantage to a busy nurse. It is made of fine lawn, and may be trimmed either with Valenciennes lace, plain frilling, or Cash's lace-edged frilling. A shape which always looks neat is the "Sister Dora," and the new pattern now being shown is especially so. It is made of washing cambric or fine linen, and will wash as easily as a pocket-handkerchief. Every variety of taste with regard to cap-strings is catered for, and some very pretty drawn-work has come from the West Indies; these are samples, and the stock is just coming in. One which will be found especially fascinating has a tiny edge of drawn-work and five little tucks. In nurses' cloaks there is an extensive choice, both of pattern and material; they may be had in a severely plain style, fitting close to the shoulders, or frilled and smocked according to fancy. The materials used are a fine all-wool cravenette Cashmere cloth, perfectly waterproof, in black, navy blue, myrtle, grey, and brown; a fine serge, thoroughly shrunk, or, for winter wear, in all wool, melton or army cloth, or Cheviot serge. The "Cecil" is a plain close-fitting shape, with full sleeves; another useful shape is the "Clarinda," a cloak with detachable cape, which may be lined with cardinal, navy or black Surah silk. It is made in several varieties of cloth, for summer and winter wear, and all Messrs. Garrould's cloaks are guaranteed all wool and thoroughly shrunk. The prices are wonderfully moderate—from 23s. upwards—and the quality is really excellent. There is a neat coat and skirt for bicycling, to be worn with the uniform bonnet, or, if preferred, the cloak may be worn, in the form of detachable skirt and cape. Messrs. Garrould have sent out an excellent assortment of patterns of uniform materials, both for dresses and cloaks; their strong Galatea washing twills, in various colours, and at exceedingly moderate cost, will be found splendid for hard wear in the wards or the district. Another very serviceable material is the Milo gingham, also quite cheap and strong. A pretty light blue or pink dress of linen-finished Halifax cloth, made expressly for nurses' wear, always looks well; it may also be had in navy blue, red and grey. A grey shrunk coating, 48 inches wide, would make a neat bicycling coat and skirt for summer use. I must not omit to notice the district bags for nurses; they are made in cowhide, and are calculated to resist the daily wear and tear of a district's nurse's work. A more expensive kind is made of real morocco, but fitted or unfitted the price is not to be complained of, and the contents of the fitted bag seem literally endless. Nurses should send for the "Red Cross Catalogue" of nursing requisites, and keep it by them for reference; while those living in London would be wise to go and examine this truly fascinating collection for themselves. The tea-room will be found a very convenient meeting-place for friends.

### MESSRS. EGERTON BURNETT.

A number of patterns have reached us from Messrs. Egerton Burnett, Wellington, Somersetshire, and so varied in assortment are they that the task of selection is a very difficult one. There is something to suit all tastes, and indeed all purses, from superior serges of soft finish at about 5s. a yard, down to sweet muslins for less than 7d. The coat and skirt, for out-of-uniform wear, is always convenient and becoming, and nurses on the look-out for a new summer dress cannot do better than send for patterns. The "Royal" serge at 2s. 6½d., 54 inches wide, is excellent in quality, and can be had in various colours, as well as in black and the ever-useful navy blue. Then there is the "Dorcas" serge, very strong, for 7¾d. narrow width, in black, navy blue, and colours. It is marked "for benevolent purposes," but there is no reason why an inexpensive dress for everyday wear should not be made of it, and it would wear splendidly, like the "school-boy serge," which is calculated to resist rough usage. For cycling, there is a very nice serge in quiet colours, very wide, for eighteenpence the yard; either a grey, green, or brownish shade would look neat, and would not show the dust. Then for better costumes, the "Loxley Coating" is excellent, and so is the new panne cloth—so soft and delicate to the touch; or, for a dress with bodice, the "Marlborough," in very delicate shades, would make up admirably. For washing dresses there is an enormous variety to select from—drills, galateas, &c.—and for blouses I strongly recommend the wonderful figured cambric at 7¾d.; it washes over and over again, and lasts from summer to summer. Muslins are always fascinating, and among these patterns are some very chaste ones in white with openwork, as well as with little flowers trailing down a stripe. For "occasions" they look as nice as anything, and if one is energetic and has time to run up a blouse it costs next to nothing. There is a particularly pretty delaine. It has a white satin stripe, with a delicate design of rosebuds thrown across it; the rosebuds are made in several colours, though pink and green are the daintiest. The zephyrs are bewildering in their variety. The "Royal stripe," figured silk, and checks, would all make pretty blouses, and there is one, a "useful" zephyr, which is only 6¼d. a yard. Shrinknaught flannel for cooler days is a very good and economical material, both for its wearing and washing qualities. I was almost forgetting to mention the silks: for half-mourning there is a very neat grey Japanese silk, either plain or figured, and there are washing silks in large variety and at very reasonable cost. For under-skirts, the "elegantis skirting" will be found excellent.

So far I have dealt only with out-of-uniform dresses, and I hope that what I have said will help some nurses who are wondering what it would be wise to purchase for wearing on a visit or during the summer holiday; as a rule they have no desire to go to great expense, so I have had the average hardworking and economically-minded nurse in mind in preparing these comments, since she forms by far the largest proportion of the profession.

And now a word about uniforms, both indoor and outdoor. I have submitted the patterns of galateas and serges to several nurses, since they know by experience what washes and what does not, what shrinks and runs, and what does neither. And they have nothing but praise for Egerton Burnett's materials; and the fact that the uniform dress material of the Queen's Nurses comes from Wellington speaks for itself. Nothing could be stronger or more wear-resisting than these galateas; they are in plain colours, broad or narrow stripes, or with spots on a plain ground; and the price is very moderate. Then for cloaks there is a choice of waterproof serge, which can be, if desired, made by the firm at reasonable cost, according to measurements sent by post. A special notice reminds intending purchasers that, except in the case of materials always in stock, it is as well to make a second choice in case the first is sold out; this, of course, does not apply to uniform materials, which are always in demand, but to those more ephemeral patterns, of which I have spoken before.

### A Book and its Story.

### THE FIRST GREAT ENGLISH KING.*

"ALFRED is the most perfect character in history. . . . No other man on record has so thoroughly united all the virtues both of the ruler and of the private man. In no other man on record were so many virtues disfigured by so little alloy. A saint without superstition; a scholar without ostentation; a warrior all of whose wars were fought in defence of his own country; a conqueror whose laurels were never stained by cruelty; a prince never cast down by adversity, never lifted up to insolence in the hour of triumph, there is no other name in history to compare with his." This eloquent tribute, from the pen of Professor Freeman, to the memory of the great and good king forms a fitting introduction to a notice of Mr. Macfayden's fascinating and scholarly record of his life and times. No recent work on the subject could be more attractive. "It is impossible to write history without rethinking it," with the result that individual records cannot fail to be influenced by the mentality of the writer. Given, as in the present instance, sound scholarship, far-seeing intuition and breadth of view, with a facile literary style, the effect cannot fail to be happy. Even if one had not read the modest opening Apology, which explains the origin of its authorship, it would be easy to divine that its writing had been a labour of love. Every quotation, every sentence, suggests it. There is a harmonious sequence of ideas and a virile force running throughout, giving vitality to each chapter, that proves it. No one not entirely in touch with his subject could write so felicitously. The incapacity of a friend through illness to produce the Life of King Alfred for the "Saintly" series in time for the millenary celebration of his reign was the cause of Mr. Macfayden's undertaking it. He says: "Written under the depressing weight of work, amid squalid and sordid scenes, under anxieties unusually oppressive, it has been a pleasure to me, beyond my own expectation and power to explain, to find myself at the beginning and end of the day in Alfred's strenuous and inspiring company; to forget the burdens of a present warfare in watching Alfred wage his; to see him battling against enemies within and without, and compelled, in the interests of order and truth, to wrestle for the mastery as much with his own friends as with the untoward circumstances of his time; and to recall how he gave

'to meanest issues fire of the Most High.'

Though the book has been confessedly a big work . . . it aims at being faithful to the data accepted by the best-informed and most recent students of King Alfred's time and work. It makes no pretension to expert Anglo-Saxon scholarship, but it is hoped that it may be found scholarly in the larger sense of discriminating between truth and legend, and of stating the facts of Alfred's life in an order and proportion which should make the whole narrative neither false nor unfair to the impression left by the great king on his own and subsequent generations." This aim has been conscientiously fulfilled by the author.

The childhood of Alfred and events contemporary with it is not the least interesting part of the narrative. There is a passing word on his mother Osburh, daughter of Blac, the King's Cupbearer—an office of high rank among West Saxon nobles—described as a noble woman by birth and nature, and religious also. It is especially interesting in connection with her name to observe the high position women held in those days among the Saxon thegns. "We may recall that in no country in the world of the ninth century did woman take higher place than with them." Tacitus records, even before Christianity had cast its halo round the head of womanhood, "The Germans believe that the sex has a certain sanctity and prescience, and they do not despise their counsels nor make light of their answers." "They were admitted into that comradeship in council and in home which comes from sharing the perils of war."

Alfred was born in the year 849. "In the year 853, King Ethelwulf not being able to go to Rome himself, despatched Alfred, in charge of the Bishop of Winchester, accompanied by a worthy escort of nobles and commoners to carry his gifts and prepare the way for his own coming." The bishop bore the name of Swithun, later to be canonised as the Saint of Showers. Youth, it said, "is the nursery where the future is always growing." And this visit, short though it was, is supposed to have been an active factor in the shaping of Alfred's life. "His residence there may be taken into account in explaining that love of literature and sound learning which led him afterwards to translate and edit for his own people what he regarded as classics in science and literature and religion of that age. He could hardly fail to contrast what he remembered of Rome with what he found in England; and he was one of those who cannot see a great want without trying to remedy it." When at the Papal Court he won the favour of the Pope, as elsewhere, and was by him anointed future King of the West Saxons, although at this time he was several steps from the throne, being himself the fifth son of Ethelwulf and Osburh. On his way home a visit to anothe foreign Court, that of Charles the Bald, King of the Western Franks, whose daughter Judith his father married as second wife, had its lasting influence on his mind. "Here he had an early introduction to the ways of the greatest and most famous Court in Europe. . . . He could never again shut out from his view the great world beyond his own kingdom. . . . In three months' stay at this Court, Alfred caught a glimpse of the best traditions of mediæval monarchy. . . . If Alfred's views of a good king are to be understood, they should be traced back to their fountain head through Charles the Bald, Lewis the Pious, to Charles the Great."

"The external features of dealing are made for us; the dealing of the inward man is determined by himself; and to the majority of men the formation of the inward man comes in the war of sense and soul. . . . It is clear that Alfred had been hampered from his childhood by some violent and troublesome disease, probably epilepsy." In the troubled, disturbed times in which he lived this thorn in the flesh must have made his kingly position doubly difficult. "There are few tasks more difficult than to take up heavy burdens while struggling with physical ill-health. . . . But Alfred was one of the thousand able to battle against disorder within and without at the same time." He married at the age of twenty, Ealhswith, the daughter of the Earl of Gainas, whose name survives in that of the town Gainsborough."

A picturesque summary of the Great King's closing years is given. "The sun of Alfred's day set in a great peace . . . the next two years, 898, 899, are memorable for the felicity which has no history. . . . We may think of the much-tried king, weather-beaten, storm-tossed, passing out of the storm and stress of conflict into the quiet rest of these years, watching his much-loved and well-served people enjoying some fruits of his labour; maturing in himself and in his kingdom the plans he had conceived and nursed with steadfast patience, seeing of the travail of his soul, and being satisfied." The whole book, whether treating of domestic, military, political, or ecclesiastical matters, teems with interest, and the author has drawn his information from the best and most authentic sources.

* "Alfred the West Saxon, King of the English." With illustrations. By Dugald Macfayden, M.A. (Publishers: Dent and Co., London. Price 4s. 6d. net.)

### District Nursing in Spain.

By a Correspondent.

One could hardly find an equivalent for "district nursing" in the Spanish language; certainly not the reality as we know it in our enlightened country.

It having fallen to me to spend some time in the fair town of Port St. Mary on the Andalusian coast, I happened to see something of the distress and misery where sickness had penetrated behind those picturesque white walls to an unsanitary interior.

I often accompanied my mother (one of the few "nurses born, not made") in her self-imposed round of district visiting, and there was not a cottage or tenement where "Doña Isabel" was not received literally with open arms by the warm-hearted folk. One bright afternoon as we were strolling up and down the housetop and watching the storks feed their young on the cathedral tower across the square, one of the servants came hurrying up with bad news written on her breathless and rather swarthy countenance.

The mother of Salvador was in one of her trances! All here are known by their Christian names, and the said Salvador was a young student in very poor circumstances, whose invalid mother, well known to us, suffered periodically from cataleptic fits.

Not knowing whether the doctor had been summoned we put a few necessaries together, threw on our mantillas, and hastened down the cobbled streets (through which, in the more slummy quarters, the open sewers run freely), shading our eyes with our fans, for the turquoise blue sky and white houses lit by the sun make a glare which is dazzling. Turning down "the street of peace"—inappropriately so named, inasmuch as it has six taverns always full of brawlers—we stopped before a stout wooden door which stood open, and entered a cool stone-flagged court, gay with flowering cacti in pots, without further ceremony.

### The Patient.

We received a pleasant "Buenos dias" from the occupants of the first flat as we climbed the wide stone steps, and the unspeakable odour of garlic followed us from their kitchen.

Reaching the second stage we pulled the bell-rope hanging outside a tall door, half glass, the framework painted white. We were admitted by a very pretty Spanish girl, a rose in her sleek black hair, but her apron to her eyes. "Thank the saints, Señora!" she exclaimed, flinging her arms about my mother's neck. "Have the goodness to come this way." We followed her down the tiled passage; it had a bright outlook on to the court which was full of flowers in pots including the tall graceful leaf of the plaintain. She opened a door, and led us through a tiny kitchen, also redolent of garlic, to a bedroom which was a sitting-room and sacred to the use of the student brother; through that again, to a windowless bedroom, only lighted and ventilated by the outer apartment! The room was so full that we had some difficulty in finding the patient.

She was lying tense and rigid, propped up by a chair behind her pillows; her head was strained back, and she breathed stertorously, as though under an anæsthetic. The doctor, a tall courtly Castilian, who, fortunately for his patients, had studied in France and America, turned to my mother with a smile. "A la buena hora!" (in good time) he said, bowing, "I shall now leave her in your charge, Señora." He drew her aside, and explained that the patient would probably recover consciousness shortly, the fit having been a long one, and he should be glad if she could clear the room of people. "It is impossible to alter her position," he said, glancing at the patient as she lay perilously near the edge of the bed; "any attempt to do so by force and we might snap the bones, Señora!" He promised to return in the evening. "One day and she will have one of these fits and never return," he said, shrugging his shoulders, but sympathetically, as he went away.

### Nursing Under Difficulties.

It was a difficult matter which he had delegated to us with the selfishness of man. Such a Babel! All talking in their high-pitched Spanish voices; some weeping and wailing out dismal forebodings: "May the holy Mother of Sorrows have pity on poor Carmen here! So soon motherless! Ay! Ay de mi!" and a noisy tearful embrace for "poor Carmen" who wept yet more bitterly. While I coaxed out the last reluctant spectator, my mother stood by the bed rubbing the upturned throat and chest with Eau de Cologne. The patient spoke occasionally in a hoarse, unnatural voice. When my mother stood by her, she began to wander in French. Presently she began to breathe in long, sobbing gasps, light returned to her glassy eyes, and she became conscious and greatly agitated.

Carmen brought some soup-jelly heated up, and served in a quaint bowl of blue and green earthenware, and we fed and warmed her—the doctor having forbidden stimulants. We had already opened the windows of the next room to admit indirectly the sunny air, spicy with the scent from an orange garden not far away. When more restored, "Seña" Margarita was interested to hear that she had been puzzling the neighbours by her French. "I lived at Marseilles when a little one," she said, "and it must have been when you came in, Doña Isabel, for I am sure I had a sense that you were here before I woke."

This was my first experience of a cataleptic fit, and a very superior specimen of a Spanish sick-room, even amongst the better classes. Alas! that a friend in need in the shape of a modern district nurse would but shock their ideas of propriety, unless she could secure the permanent attendance of a *chaperone* in her round of visits!

### Appointments.

Bradford Children's Hospital.—Miss Grace Horsman, has been appointed charge nurse. She was trained at the Clayton Hospital, Wakefield, and has since been nurse at the National Hospital for the Paralysed and Epileptic, Queen's Square, Bloomsbury, and sister at the Royal Halifax Infirmary.

Chalmers Hospital, Edinburgh.—Miss Junor has been appointed charge nurse. She was trained at the Northern Infirmary, Inverness, and has since for four years been charge nurse in the fever wards.

Cottage Hospital, Stranraer. — Miss Margaret Cameron has been appointed matron. She was trained at the Western Infirmary, Glasgow, and has since for eight years been charge nurse of the medical ward at the Northern Infirmary, Inverness.

Fever Hospital, Chester-le-Street.—Miss S. M. Yeates has been appointed nurse-matron. She was trained at the South Devon and East Cornwall Hospital, and has since been engaged in private nursing at Newcastle-on-Tyne and elsewhere.

Market Drayton Cottage Hospital.—Miss L. L. Rose has been appointed matron.

Northern Infirmary, Inverness.—Miss J. Ker has been appointed charge nurse of the surgical wards and theatre. She was trained at Cumberland Infirmary, Carlisle, and has been since charge nurse at Beckett Hospital, Barnsley. Miss Amelia Kennedy has been appointed charge nurse for the fever wards. She was trained at the Northern Infirmary, Inverness, and for the past four years has done private nursing on her own account.

Plymouth Workhouse.—Miss Eliza Cage has been appointed assistant nurse. She was trained by the Meath Workhouse Nursing Association, and has since been assistant nurse at the Hastings Union.

Royal Berks Hospital, Reading.—Miss Martha A. Garner has been appointed sister. She was trained at the General Infirmary, Leeds, and has since been sister at Grantham Hospital.

Royal Halifax Infirmary. — Miss Evelee Dane has been appointed night superintendent. She was trained at Western Hospital, Glasgow, and has since been charge nurse at Longmore Hospital, Edinburgh, charge nurse at Tooting Fever Hospital, and night superintendent at Wolverhampton General Hospital.

### Ward Washing Day in a Bombay Hospital.

By a Sister.

Most of the hospitals in the Bombay Presidency have tessellated or asphalte floors, but we have not yet risen to the height of such a luxury in our little hospital. The upper storey has a boarded unpolished floor, and there are numerous large cracks—the lower floor is flagged. The washing of these wards is consequently quite an undertaking, especially when the fact is taken into consideration that no native understands dry-scrubbing. His one idea is to swamp the floor with bucketsful of water; elbow-grease is quite a secondary matter. To get natives to grasp the use of a swab requires a long course of training and the expenditure of much patience. The lower floor fares the worst in the matter of washing, for when the wards above are washed the water streams down into the lower ones through cracks innumerable.

### The "First Surgical."

We have fixed days on which the several wards are washed out; on the lower floor, for instance, supposing it is the day for the first surgical to be washed out, directly the surgeon's visit is over, the nurses, ward-boys, and attendants set to work to clear the ward. Fractures, liver abscesses, recent amputations, are carried out on their beds to the front verandah. The bedding of the remainder is spread out in the verandah, and they get out on crutches, or otherwise. Every cot, the occupant of which is able to be moved, is taken into the back compound, there to be well beaten, and, if possible, taped afresh. The bedsteads are iron frames, taped closely and tightly with thick webbing; the bolts and screws are painted with carbolic acid, or kerosene. The safes and stools are carried to the tank and scrubbed; when dry they are oiled to keep away ants; the tables, mackintoshes, caseholders, are all polished. As many of the patients who are able to do so ask to be allowed to help, and seem to thoroughly enjoy it. The ward-boys bring in buckets of water and their scrubbers, and with a great deal of noise the washing progresses. The mid-day meal has usually to be served while the patients are out in the verandah. Those who are confined to bed seem to relish this "outing;" the ward is generally settled in again by 1 P.M. Each patient may bring in his or her own eating and and drinking vessels, otherwise the hospital supplies each with the following: a thali (shallow round plate), a lota for water, and a cotora for soup, &c.; these are all made of brass, and the ward boys are held responsible for their cleanliness and polish. Our feeders at present are of tin.

### Unwelcome Visitors.

Every morning and afternoon a strong solution of carbolic is swabbed underneath all the beds. A constant warfare is waged against bugs; for one thing, the visitors bring them in; and then serious cases are often admitted during the night, and have to be put to bed just as they are, as far as washing and changing them goes, for the night nurse has not time to do this. Accordingly before morning the bed may have many occupants. A patient once complained to me about the number of bugs; I told him that every means were taken to keep them down, but that visitors brought in so many. That very afternoon his mother came to see him, and while she was crossing the ward to his bed, I saw one of the objectionable animals walking over her white urneé (a long sheet which Mahommedan women often wear when going out). I went over to the bed at once and pointed it out to them both; I heard no more complaints from that quarter. This is only one of frequent experiences of the kind.

### Natives' Affection for Insects.

Many castes will not kill any animal or insect, bugs included. Once when riding in a tram-car in Bombay five native men were sitting about three seats away from me, and there was a large bug walking over the sleeve of one of them. His companion quietly picked it off, and landed it safely on the floor. Although I had not long been seated, I stopped the tram and finished my journey on foot. Some castes go so far as not even to kill snakes. They set traps for them, then take them out into the jungle and set them free. Up country there is a particular caste who wear a piece of cloth over their mouths, so that no insect is killed by being swallowed, or by the heat of their breath. With all these difficulties in our way, it will be easy for you to comprehend the colossal attempts necessary to keep under, to some extent at least, the bugs in an essentially native hospital.

### A Nasty Habit.

These people are very fond of secreting small pieces of filthy rag under their pillows, or in their safes, so that every day a certain number must be inspected. The nurse, too, often finds "bidies" (a native smoke) in the locker belonging to a patient for whom smoking is prohibited. The rule as to smoking is that all patients able to leave their beds must smoke in the verandahs or compounds; those confined to their beds must not smoke until after the doctor's visit; there must be no smoking by anyone after the house-surgeon has visited at night.

### Smuggled Opium.

In the native women's ward the nurse has constantly to be on the alert for smuggled opium for the babies. I have seen babies belonging to well-to-do natives who have always had their small amount of opium daily quite strong and healthy; but those belonging to the poorer classes, where a large amount is given to keep the child asleep while the mother goes out to work all day, and where it takes the place of nourishment, are thin and pinched, dull-eyed, and altogether miserable, pitiful objects. For every native child that we have in hospital, to the age of about eleven or twelve, the mother or father insists on staying in too, so that we have no children's ward. Most of the parents are very willing to help, and we are always so short of hands that I have often thought them a welcome evil, though at times they are a great worry.

### Madonna of the Wards.

'Tis but an unstrung lute I take
Madonna of the wards!
To bid a long, sad silence break
In sympathetic chords,
Which shall, upon their trembling crest,
A song of friendship bring;
That now is locked within my breast,
Mute, and uncomforting.

. . . . . . .

Fresh, joyous, dainty as a rose
Bathed in the dews of morn,
About her work she deftly goes,
Ere hardly day is born.
O! cheering glance; O! question kind.
One fain would kiss the hem
Of raiment spotless as the mind;
Fit casket for the gem.

Sweet voice, calm eyes, cool gentle hands,
Held out in sympathy,
To ev'ry creature that demands
Her tender ministry.

The aching limb, the throbbing wound,
Find, there, a soothing balm:
While, to the fevered mind attuned,
Her counsel breathes a calm.

When dire the day, and sad the hour;
When weary wears the night,
And, restless, haunting phantoms glower,
With her there comes the light.
And children, dreaming of the slum,
Wild terror in their breast,
To her enshielding haven come,
And, on her bosom rest.

Though knowledge shall have claimed the place
Whence ignorance has fled;
Yet, in her soul as in her face,
Bright purity is read.
If she have probed the villain's heart,
Has seen the pauper's crust,
There lies beneath the angry smart,
In God her perfect trust.

And, though through life stern duty set
A course for her to sail,
Her friendship lingers with me yet,
Till time and memory fail.
A woman planned by God to be
A comforter and friend;
Wise, simple, gentle, fragrant "she,"
Lo! at thy shrine I bend!

ARTHUR FROST, *Surgeon.*

### Everybody's Opinion.

[Correspondence on all subjects is invited, but we cannot in any way be responsible for the opinions expressed by our correspondents. No communication can be entertained if the name and address of the correspondent is not given, as a guarantee of good faith but not necessarily for publication, or unless one side of the paper only is written on.]

### THE QUEEN AND THE ROYAL NATIONAL PENSION FUND FOR NURSES.

"A POLICY-HOLDER" writes: With reference to E.F.'s letter I have sent a suggestion to the secretary of the R.N.P.F., as I feel sure a special request to Their Majesties—our present King and Queen—for a new design with the coat of arms would have Their Gracious Majesties' due consideration. As a policy-holder I wear the armlet regularly, as I find my patients admire and appreciate the honour of my being termed a Princess Nurse. Yet, what is in a name except a nurse proves of really more value than that at first put upon her?

### PRESENTS TO NURSES.

"NURSE NO. II." writes: Do patients or their friends think it almost compulsory to add a slight present to the stated fee of a private nurse? I think not. But, certainly, I should not take it as an insult. Surely it lies in the nurse's power to accept or reject a present offered, often out of pure gratitude. Should she choose to accept, it may be done gracefully, and it will in no wise, I think, lower the estimation of herself or the moral standard generally. Equally, she may reject a present on principle or for any other reason. Therefore I think it a case where no hard-and-fast rule can be laid down, as it is a matter for nurses to settle individually.

### THE CROYDON CRISIS.

"DEEPLY INTERESTED" writes: I wish I could think it likely that all nurses would be so loyal to their profession that not one would apply for the post now advertised by the Croydon Board of Guardians. Miss Julian has had my sincere sympathy throughout. When I was a subordinate I used to wonder how it was that there were so many unprincipled and unreliable nurses, and why superintendents and matrons permitted untrustworthy probationers to remain. I understand their difficulties better now, for I have been unsuccessful in my attempts to weed out unsuitable candidates, and when their certificates come to be signed I may find myself in the same position as Miss Julian is now. The attitude of the Local Government Board is difficult to understand. Surely Miss Julian deserves the gratitude of the public and of all right-thinking nurses for trying to prevent the admission of unworthy members into the profession.

### ACTION BY A NURSE.

"SISTER FLORENCE," matron of the Diphtheria Hospital, East Ham, writes: I beg to inform you that the whole statement of the case in the "Action by a Nurse" in your last week's issue of THE HOSPITAL is absolutely untrue. A clear and definite business arrangement was made with Nurse Frost at a personal interview on February 13th. She was taken round the hospital and everything told her on that occasion. As she was unable to come at once, and as we were short-handed owing to a nurse being ill, a private nurse was engaged for the time being to keep the post vacant for her until February 25th. She was engaged from 151 Hydlethorpe Road, Clapham Park, S.W., not from Wisbech, and arrived at the hospital at 9.20 P.M., not 8.40, on February 25th. She was interviewed by the charge nurse. The rules were certainly not given to her; they were hanging in her bedroom. The following morning tea and hot water were taken to her at 6.30, she got up at 8, went to the wards at 8.20, left them for two hours' leave at 10 A.M., was seen by me at 10.15 A.M. Nurse Frost was not reprimanded for coming late the night before, for no time had been specified for her to arrive, but her manner was very rude and resentful when she was asked why she did not get up when called. She also objected to sharing her bedroom with two other nurses; owing to this fact she was asked if she quite understood that she was only engaged a month on trial, and that if she did not like it she could leave then. She immediately replied "No;" that she would not stay on those conditions; that she was not a probationer, but would go at once and say she had been sent, also claim a month's salary in lieu of notice. She thereupon left the room and the house. Such are the facts of the case: whether they are believed or not matters very little, but I should like to ask what object would any matron have in discharging a nurse for no apparent reason?

"S. C. B." writes: I wish to correct a statement in your columns regarding Miss Frost's action against the East Ham District Council. She entered the hospital as staff nurse on February 25th, and arrived at 9.20 P.M., and as matron was not on duty I, as matron's assistant, received her and ordered her supper. She was then shown to her room. Certainly the rules were not handed to her, for they were hanging in the bedroom. She was told she would be called at 6.30 A.M. for breakfast at 7 A.M., to be on duty 7.30; and though she was called (and a cup of tea given to her), with the other nurses, she did not get down to breakfast till 8 A.M., and went on duty about 8.20. As the night nurses had done the first part of her duties, she was told to comb nine children's heads, which was the "disagreeable task" mentioned, and why that should be termed "disagreeable" I fail to see, for it is quite as essential to attend to the personal comforts of the patients as to treat their ailments; and who should instruct a nurse as to what duties to perform if not the charge nurse? She objected to sharing a bedroom with two other nurses, and her whole manner was very resentful on first entering the hospital. She left the wards at 10 A.M. for two hours' leave, and, meeting another nurse on the stairs, informed her that she (Nurse Frost) was "going then," as she did not care for the place, and she left the hospital of her own free will.

[The report of Miss Frost's action was summarised from the daily papers, and, so far as we are concerned, no mistake was made.—ED. HOSPITAL.]

### SO-CALLED TRAINED NURSES.

C. LUCIUS THOMAS, assistant masseur, Imperial Yeomanry Hospital, Deelfontein, writes under date of March 3rd:—I notice that matters discussed in the column headed "Everybody's Opinion" are generally done with in a week or two and we see no more of them. It is therefore possible that the above subject will have been allowed to drop, and that this letter, coming from so far and taking a long time, will by the date it reaches THE "NURSING MIRROR" be out of the running. Those who are afraid of the light will probably, as usual, leave it severely alone for fear it might show them up, while those like "A Male Nurse and Masseur," writing in your issue of February 2nd, only just to hand, will also for a different reason wish it to be hushed up—the one for fear of exposure and consequent loss of income, the other for fear of injury to himself and good men generally who are male nurses. A nurse (man or woman) ought to sacrifice his or her own interest in the cause of humanity and the good of their fellows. But when the ideal is merely to make money anyhow, it is high time that the sacredness of life, and the public in general, should be protected against such people, who justly deserve to be termed charlatans. The case quoted is not (as your correspondent says) an isolated case, for I know that men are sent our to nurse who have not in some cases had more than one year's training (so called), usually meaning that they have spent that time in an asylum, where they may have seen paralytic seizures, epileptic fits, and the bedsores of general paralytics. They may also possibly have seen an enema given and a catheter passed once or twice, perhaps not. In any case they are prepared to do anything and everything provided there is a fee attached. Well, a telegram comes to the "Institute" to send a male nurse, sometimes indicating the nature of the case, more often not. This man is sent, and he carries a letter of advice. If there is anything to indicate that the case is a mental one the letter is filled in something like this: "Attendant has this day been sent," &c., the idea being to convey to the doctor or employer that the man is specially suited for that particular case. If, again, there is anything to indicate that it is a case for a hospital nurse it is called a "nursing" case, and "nurse" is put on the paper and sent, the motive being to impress the people that he has had special training, and is prepared to do anything from washing a gentleman's hands and face to preparing for and attending a trephining, a colotomy, or any other case. This sort of thing has been, and is being, done by the so-called superintendents of male nursing institutes advertised under various names in London. They are usually proprietary concerns run by one individual assuming the title of association, or of late years co-operation. My object in writing is not to bring a charge against anyone personally, but in the hope that someone who has the power will regard it as a beckoning finger pointing in the direction of one of "the wrongs that need resistance" and to "the cause that lacks assistance," by which I mean the men who are qualified and are trying to form for themselves a genuine co-operation by means of which the outrageously exorbitant charges made by the proprietors of such places as I have mentioned would be avoided and the deserving men get what they work hard for, while the untrained, inefficient, and unsuitable men would be rigorously and unrelentingly debarred from practising. I came out here in June last. I am glad to say that I have the honour of being assistant masseur to a grand and noble institution like the I.Y.H., which initials are placed (in white) on the side of the "kopje" overlooking the hospital, and each letter is over 30 yards (actual measurement 95 feet) long. The chief masseur has also a knowledge of hydropathy, and has erected a Russian bath (steam from the laundry), and also later a fine plunge bath 8 feet 6 inches square, 6 feet deep. It is really wonderful what has been done here. The baths are a veritable God-send amidst the sandstorms and heat. As regards massage, the report of work done here must prove very interesting when it is (as I suppose it will be) published.

### Where to Go.

LECTURE by Dr. Tom Robinson on "Short Cuts" at the Trained Nurses' Club, 12 Buckingham Street, Strand, Friday, April 26th, at 7.45.

### Wants and Workers.

A NURSE will thank any kind friends who will send old linen for a poor old man suffering from cancer. Tompsett, Wick Street, Firle, near Lewes, Sussex.

### For Reading to the Sick.

### THE SOUL'S DISCIPLINE.

LIE still my restive heart, lie still:
God's word to thee saith "Wait and bear."
The good which He appoints is good,
The good which denies were ill.
Yea, subtle comfort is thy care
Thy heart a help not understood.

"Friend, go up higher," to one; to one
"Friend, enter thou thy joy" He saith:
To one, "Be faithful unto death."
For some a wilderness doth flower,
Or a day's work in one hour is done,
"But could'st thou not watch one hour?"

*Christina Rosetti.*

Illness is not a cessation of life, but a different life, which has its own ideals, its own duties, its own opportunities, even its own amusements. It is a chapter of your life, which has just as much meaning in it as any other; when it closes, by death or by recovery, you may be able to see that it had even a deeper meaning—that is, if you live it "with intention,' not if you slip through anyhow, from day to day.

Arrange your life—on all days when it is possible, keep to your plans; when extra pain or weakness interferes with them, accept it as God's own re-arrangement, not as a disturbance to be fretful over, or, as a reason for losing heart in your efforts at regularity.

If you are going to recover, do not let slip such a golden opportunity as this illness affords you of living perfectly in a detached bit of life.

This illness is a definite, distinct chapter, which you can illuminate in letters of gold if you will. In one way it is a hard chapter, in another it is easy, because its very hardness makes you brace yourself only to the highest source of help.

Perhaps you are not going to recover, but must face a life of limitations and of exhaustion, worse to bear than any actual pain; then hear Epictetus saying, "Everything has two handles—one by which it can be borne, one by which it cannot be borne.

Take up your "spoiled" life by the right handle, and make it a beautiful life, by living it "with intention." It is a "day of small things," but they can be done in a large spirit, which will make them part of Eternity. Obedience and gladness will be the atmosphere of Heaven—make your sick-room an ante-chamber of Heaven, by virtue of sharing in that atmosphere.—*Lucy Soulsby.*

When the shore is gained, who will heed the toil and the storm? And we shall steer safely through every storm, so long as our heart is right, our intention fervent, our courage steadfast, and our trust fixed on God. If at times we are somewhat stunned by the tempest, never fear; let us take breath, and go on afresh. Do not be disconcerted by the fits of vexation and uneasiness which are sometimes produced by the multiplicity of your anxieties. No, indeed, dearest child, all these are but opportunities of strengthening yourself in the loving, forbearing graces which our dear Lord sets before us.—*Francis de Sales.*

And the heart shall find repose,
Time to tell life's tale of blessing
Ere the solemn shadows close;
Time to take a loving survey
Ere the well-known landscape fade,
And the call to come up higher
Shall be silently obeyed.

*A. R. Coudin.*

### Notes and Queries.

**The Editor is always willing to answer in this column, without any fee, all reasonable questions, as soon as possible.**

**But the following rules must be carefully observed :—**

1. **Every communication must be accompanied by the name and address of the writer.**
2. **The question must always bear upon nursing, directly or indirectly.**

**If an answer is required by letter a fee of half-a-crown must be enclosed with the note containing the inquiry.**

*Malta Fever.*

(20) Will you kindly tell me the chief symptoms for which to watch in nursing a case of Malta fever?—*Private Nurse.*

All the symptoms must be watched. Malta fever is often a tedious affair, and the tendency to relapse is so great as to form one of the main features of the disease. The nurse therefore must not speak with any confidence in regard to the corner having being turned on the strength of the temperature having gone down to normal. The nursing is of course that of fever-nursing during the febrile periods, and great care in diet is afterwards requisite, no solid food being generally allowed until fever has been absent for at least ten days. Common complications are swelling of the joints, profuse sweating much like that of rheumatism, and debility and anæmia.

*Excrement Destructor.*

(21) I saw a "typhoid excrement destructor" mentioned in The Hospital Mirror some time ago, will you kindly tell me where I can obtain information respecting it?—*J. J. T.*

If J. J. T. will refer us to the article in which this apparatus was mentioned we shall be better able to answer her question.

*Hypnotism.*

(22) Is there any place in England where a patient suffering from mental depression and hysteria could be treated by hypnotism?—*Nurse Jean.*

Any medical practitioner who devotes himself specially to hypnotic treatment would undertake such a case. It is not so much a matter of place as of person, and in regard to this no doubt your doctor could advise you.

*Vacancy.*

(23) A short time ago I applied to enter a hospital as probationer. Sending me some papers to fill up they informed me that there would not be a vacancy before the spring. Ought I to expect a reply before there is a vacancy?—*S. P.*

No. It may be some months before there is a vacancy. You might write asking when one is likely to occur, enclosing a stamped envelope for reply.

*Training.*

(24) When I am old enough I want to train as a nurse in a large London hospital. Would I stand a better chance of acceptance if I had some previous training? (2) Are fever cases nursed in the hospitals for general nursing? *Country Lass.*

Matrons, as a rule, prefer probationers well grounded in domestic duties and ignorant in nursing. (2) Isolation wards or cottages are attached to the large hospitals, but infectious cases are, when possible, sent to isolation hospitals.

*Supplementary Training.*

(25) Can I get short additional training, with salary, in surgical nursing. I am a maternity nurse with some years' experience in nursing delicate children?—*Nurse.*

You might hear of something that would suit you by means of an advertisement.

*Probationers.*

(26) Does the Nottingham Children's Hospital train probationers from the age of twenty, and which is the best children's hospital to enter, London excepted?

What salary should I receive?—*Molly B.*

Kindly tell me if there are any London hospitals where probationers are accepted at the age of eighteen?—*Anxious Inquirer.*

At the age of twenty.—*D. L. B.*

At the age of nineteen.—*G. M. F.*

It is not advisable to train so young as any of these, and few hospitals will accept candidates under twenty. Study the "Nursing Profession: How and Where to Train." *Molly B.* must apply direct to the Matron of the Free Hospital for Sick Children, Nottingham, for particulars as to training.

*Infirmary Training.*

(27) Kindly give names of few infirmaries where I might apply for training for three years' certificate?—*X. Y. Z.*

"The Nursing Profession: How and Where to Train," will answer your question fully.

*Training School.*

(28) I am desirous of training as a nurse. Can you advise me as to the best school?—*M. E. F.* and *M. E. H.*

Having waited, as advised, until twenty-five before seeking training as a nurse, and having always wished to be trained at St. Thomas's, London, I should be very glad of a little advice, as I am very ignorant on the subject. 2. Is there any advantage in being a "Queen's Nurse?"—*C. M.*

Is the Central London Sick Asylum, Cleveland Street, W., a recognised training school for nurses?—*N. P.*

Are there any hospitals where nurses are specially trained for private work?—*H. C.*

Please tell me if there are any general hospitals granting a three years' certificate, where probationers receive a salary from the first? Also what the salary is for the first year?—*E. C.*

1. Can I get into a fever hospital for two years where a salary is given? 2. Having only received an elementary education, am I qualified to answer advertisements for "educated probationers"?—*J. A. S.*

I am anxious to obtain one or two years' training in general nursing without paying premium. I should prefer maternity work, and I, would like to know if the training in general nursing is also necessary? 2. Can you tell me the lowest fee for maternity training?—*Stephanie.*

Is the Crumpsall Infirmary, near Manchester, a recognised training school for nurses?—*E. G. W.*

1. Is the Royal Surrey County Hospital a recognised training school for nurses? 2. Is it etiquette for the friends of the patient to remain in the sick room whilst the doctor is paying his visit?—*Inquirer.*

Having just completed three years' training in a hospital of 40 beds not maintaining a resident house surgeon, I wish to know if I could possibly get a post as staff nurse in one of the London hospitals?—*Anxious One.*

For full particulars we refer all these correspondents to "The Nursing Profession: How and Where to Train." *M. E. F.* and *M. E. H.* We do not give advice as to the best schools for training. *C. M.* You would do well to be accepted at St. Thomas's. The "Queen's" nurses are district nurses, and there is no advantage in being one unless you like to work amongst the poor. *N. P.* Yes. *H. C.* No. *E. C.* Yes, many, but the salaries vary. *J. A. S.* (2) No. *Stephanie.* General training advantageous but not absolutely necessary. (2) £7 5s. *E. G. W.* Yes. *Inquirer* (1) Yes. (2) Certainly, if the doctor permits it. *Anxious One,* advertise.

*Male Nurse.*

(29) Will you kindly tell me where men may be trained as male nurses? What training would fit a man to take any case, mental or otherwise? *C. J. T.*

How can I perfect myself in nursing? I have had four and a half years' experience. Is there any examination I could pass? (2) Is there a book which explains the use of a hypodermic syringe?—*H. C. B.*

Male nurses are trained in England in two ways. Either they enter the only hospital open to them—namely, the National Hospital for the Paralysed and Epileptic, Queen's Square, Bloomsbury, W.C., for a two years' course of instruction; or, they enter one of the more advanced asylums where probationers are prepared for the certificate of the Medico-Psychological Society.—*C. J. T.* This certificate refers to both medical and mental cases. *H. C. B.* is recommended to seek instruction in the use of the hypodermic syringe from the medical man under whom he is working; books are misleading.

*Unrecognised Training.*

(30) I have been trained for three years at a school which does not fulfil the requirements of the Local Government Board. Do you think it would be possible to enter a hospital or infirmary as assistant nurse, and at the same time attend the lectures and be prepared for the three years' certificate; or, must I become a probationer again?—*No Name.*

You can always try. Write to the Matron, Lambeth Infirmary, Brook Street, Kennington Road, S.E.

*Shortsight.*

(31) I am compelled always to wear spectacles on account of shortsight. Will this prevent my obtaining training as a nurse?—*Rex and Canopus.*

Not necessarily.

*Short Training.*

(32) Can you mention hospitals or infirmaries where paying probationers are taken for a short time?—*Inquirer.*

See the "Nursing Profession: How and Where to Train." Nearly all the big London hospitals do so.

*Maternity Nursing*

(33) I have the L.O.S. but want more practical experience. Could I get a post as charge-nurse of a lying-in ward?—*E. M. E.*

I have had eighteen months' experience in nursing, and would like to train as midwife. Is there a hospital in London which would train and pay me a small salary? I cannot afford a premium, and do not know what the training costs.—*Nurse May* and *Kathleen V.*

I should be glad of some information about training in the Rotunda Hospital (Chesterfield?)—*Asgate.*

*E. M. E.* You had better advertise. *Nurse May* and *Kathleen V.* None of the maternity hospitals train and pay salary. The lowest fees for training are £7 7s. *Asgate* must write to the Matron of the Rotunda Lying-in Hospital, Dublin, for particulars.

*Maternity Fees.*

(34) 1. What does a nurse called in to an emergency confinement case charge?

2. Is the month lunar or calendar?

3. Is a maternity nurse discharged wrongfully at the end of a fortnight entitled to an allowance for board and residence as well as her fees?—*A. M. C.*

4. What ought a nurse to take with her to a maternity case?

5. What privileges as regards meals, rest, leisure, and travelling expenses are due to a private nurse attending maternity cases? *Nurse R.*

1. The fee is regulated according to the social position of the patient. 2. Four weeks. 3. Yes. 4. The usual outfit. 5. A maternity nurse should arrange terms beforehand on all these points, convenient to herself and to her prospective patient.

*Supplementary Training.*

(35) 1. Would you kindly tell me what isolation hospital awards a certificate for six months' training?

2. Is the above period sufficient for one who has had two years' general work?—*Infirmary.*

I hold the L.O.S., and would like to get a year's general training in order to fit me for district work. I should not care to pay a premium.—*Nurse J.*

1. It is not usual to grant certificates for so short a time. Possibly a nurse already trained might come to some arrangement with the matron of an infectious diseases hospital on the subject. For vacancies see our advertisement columns. 2. It depends upon the individual. Nurse J. might be accepted and trained by one of the County Nursing Associations. See our advertisement columns, or advertise.

*Rearing Children.*

(36) Can you recommend a good book on bringing up children after infancy?—*J. Y.*

"The Mother's Help and Guide to the Domestic Management of her Children." By P. Murray Braidwood, M.D. Price 2s. from the Scientific Press.

**Standard Books of Reference.**

"The Nursing Profession: How and Where to Train." 2s. net; post free 2s. 4d.

"The Nurses' Dictionary of Medical Terms." 2s.

"Burdett's Series of Nursing Text-Books." 1s. each.

"A Handbook for Nurses." (Illustrated.) 5s.

"Nursing: Its Theory and Practice." New Edition. 3s. 6d.

"Helps in Sickness and to Health." Fifteenth Thousand. 5s.

"The Physiological Feeding of Infants." 1s.

"The Physiological Nursery Chart." 1s.; post free, 1s. 3d.

"Hospital Expenditure: The Commissariat." 2s. 6d.

All these are published by The Scientific Press, Ltd., and may be obtained through any bookseller or direct from the publishers, 28 and 29 Southampton Street, London, W.C.

### Travel Notes.

By Sister Grace.

### LXX.—MORE PALACES IN ROME.

In such a wealth of interest as the palaces of Rome place here before us, it is difficult to make a selection to figure in our limited space, but I think perhaps the Palazzo Bonaparte, formerly the Rusticucci Palace, is as interesting to us as any amongst these old Roman houses, full to the brim of romantic and historical facts and legends.

### The Palazzo Bonaparte.

It was built in 1660 by Rossi and was the scene of many a struggle for power between the Orsini and the Colonna. Here Letizia Bonaparte, "Madame Mère," as she was called, the mother of the great Napoleon, came to live, and here she died in 1836. Is it possible? I fear it is, that the Emperor was somewhat ashamed of that noble woman who to her last day could never converse in French, but retained all her Corsican ways and manners. At any rate he seemed quite satisfied that she should remain in distant Rome whilst he revelled in splendour in Paris. In this quiet Palace all kinds of domestic broils unwound themselves in the Bonaparte family.

The great man's family was divided between an intense greediness to profit to the uttermost farthing by the splendour of their head and a not unnatural kicking against that head's very summary mode of dealing with them.

Napoleon's idea was to benefit his family chiefly through powerful matrimonial alliances. His sister Caroline became the wife of Murat, and thus Queen of Naples. Joseph married a sister of Madame Bernadotte and was thus allied to the Swedish royal family. Eliza became Grand Duchess of Tuscany, and Pauline, the widow of General Leclerc, reigned in Rome as Princess Borghese. Jerome, at his brother's autocratic order, resigned his American wife and married the Princess of Wurtemburg. He became the father of "Plon Plon."

Lucien alone had pluck to resist his powerful brother's command; he twice married, but always to please himself, and at the dictates of love, thus incurring Napoleon's lasting resentment, and so it was that instead of reigning over some conquered country or petty State, he lived quietly with Madame Mère in Rome.

Letizia Bonaparte was a devoted mother, but above all her children she loved the Emperor, and she died on the camp bedstead which had been his in St. Helena and with a bust of the poor little King of Rome in front of her eyes. Mrs. Minto Elliot tells a very singular story which has obtained credence in Rome respecting the death of the Emperor, which I will give you, for it is strangely interesting.

"A strange gentleman arrived on the day and at the hour of Napoleon's death and demanded an audience of Madame Mère. He refused his name, but insisted on seeing her alone; his words on meeting her were: 'While I am addressing Your Highness, the Emperor is freed from his sufferings—he is dead. Kiss the image of the Redeemer.' . . . Some prophetic warnings of the condition of Europe in the near future followed, and he silently departed.

"Madame Mère always said that this individual bore the closest resemblance to the Emperor."

This kind, generous, devoted mother and noble Corsican matron is buried in Santa Maria in Via Lata. In this palace, too, Queen Hortense, daughter of Josephine, passed many months. She was a great favourite with the Emperor, but it did not prevent his trampling on the mutual love of Duroc and Hortense, and compelling the latter to become the wife of Louis, ex-king of Holland. Louis was at the time in love with Emilie de Beauharnais, and thus they began their ill-omened married life. Napoleon III. was the child of this marriage.

### The Palazzo Borghese.

Here we cross the family thread of the Bonapartes again. Pauline first married General Leclerc, and apparently loved him as much as it was possible for a woman of her light and shallow disposition to love anyone but herself. After his death she became the wife of Camillo Borghese; there was no love on either side—a complete marriage of convenience it naturally turned out disastrously. The Princess began to be lightly spoken of, and it is not surprising when we consider that Canova's beautiful statue of Venus Victrix is a portrait of the reckless Princess. Her life gave occasion to much scandal, but the circumstances of her marriage and the character of Prince Borghese must be considered in the light of extenuating circumstances.

### Lady Gwendoline Talbot—Princess Borghese.

The sweet memory of this gracious and much loved lady casts a gracious light throughout the vast Borghese Palace. She died in 1839 of diphtheria after forty-eight hours' illness, and all Rome followed her to her grave before the high altar in the Borghese chapel in the church of S. Maria Maggiore. At the same time the unfortunate Prince lost several children of the same complaint. The tragic death of the Princess gave rise to several curious legends, turning upon the appearance of her spirit to someone in the chapel. These stories, under the name of the Borghese ghost, appear with different surroundings. On March 13th, *The Daily Chronicle* published a short paragraph describing the story, and identifying with it the material loss of a valuable sapphire ring, which mysteriously disappeared from the hand of the buried princess. This occurred more than sixty years ago, but the memory of the beloved princess keeps any story connected with her name green in the hearts of the Romans.

### The Palazzo Cancellaria.

At this palace for some time lived Louise de Stolberg, wife of Prince Charles Edward. I fear we cannot acquit her of great indiscretion, even if there is no proof of criminality in her connection with the Marchese Alfieri. After her separation from her husband, her brother-in-law, Cardinal Yorke, must have believed in her innocence, for he gave her an apartment in his residence, the Palazzo Cancellaria. After a time, however, her conduct scandalised him, and he withdrew his protection. Eventually she lived with Alfieri in London, but there is no proof that she did so until after the death of the Prince; both Alfieri and Louise died in Florence and were buried in Santa Croce. The latest incident of interest connected with the Cancellaria is the barbarous murder of Rossi in 1848. By whom committed was never known: a secret hand was stretched out, the blow struck, and the murderer escaped, presumably by the connivance of accomplices in the crowd. Pelegrino Rossi became minister to Pio Nono and shared the unpopularity of his master. On the night of November 14th he went to see the Holy Father at the Quirinal; there he was twice warned by women friends not to return to the Cancellaria that night—they both felt a strong presentiment of his death. He laughed at them, however, and mounting the steps of the Cancellaria was stabbed in the neck; so sure was the blow that he never spoke, and was dead in less than a minute.

### TRAVEL NOTES AND QUERIES.

Innsbruck in Early Spring (Ida).—It would be delightful, and there are numerous excursions to be made. Yes, it is cold certainly, but a dry bracing cold that is very healthy. You do not say what you wish to spend. The "Tiroler Hof" is first class and reasonable. Second class "Goldner Adler," and many others. Something still cheaper is the Hotel Pension Kayser.

Jersey *versus* Normandy (M. G.)—Pseudonym too long. Expenses are about equal in the islands or on the French coast. I conclude you mean £10 each, for there are very few favoured spots where two people could live for three weeks and make a long journey for £10. If you send threepence to the Editor of *Home Notes*, Henrietta Street, Covent Garden, and ask him to send you the two travel articles of June 16th and June 23rd, entitled "A Trip to the Channel Islands," and "Guernsey and Sark," I think it will be your shortest way. In those I gave a full account of Jersey and Guernsey. When you have read them and have formed some idea of your preferences, write to me again. June is a delightful month abroad, and cheap. If you choose Normandy, the neighbourhood of Valery might suit you, but it is very quiet. If you like Brittany, the northern watering places are dear, and you would do well to choose the south, Douaruenev, &c. Let me hear again, especially as to what you wish to spend. Much travelling from place to place means greatly increased expense.

